# Collagen IV (COL4A1, COL4A2), a Component of the Viral Biofilm, Is Induced by the HTLV-1 Oncoprotein Tax and Impacts Virus Transmission

**DOI:** 10.3389/fmicb.2019.02439

**Published:** 2019-10-23

**Authors:** Sebastian Millen, Christine Gross, Norbert Donhauser, Melanie C. Mann, Jean-Marie Péloponèse Jr., Andrea K. Thoma-Kress

**Affiliations:** ^1^Institute of Clinical and Molecular Virology, Universitätsklinikum Erlangen, Friedrich-Alexander-Universität Erlangen-Nürnberg, Erlangen, Germany; ^2^IRIM-UMR 9004, Research Institute in Infectiology of Montpellier, CNRS, University of Montpellier, Montpellier, France

**Keywords:** HTLV-1, Tax-1, collagen 4, collagen IV, COL4A1, COL4A2, virus transmission, viral biofilm

## Abstract

Human T-cell leukemia virus type 1 (HTLV-1) is the etiologic agent for Adult T-Cell Leukemia/Lymphoma (ATLL) and HTLV-1-Associated Myelopathy/Tropical Spastic Paraparesis (HAM/TSP). HTLV-1 infects CD4^+^ T-cells via cell-to-cell transmission requiring reorganization of the cytoskeleton and expression of the viral transactivator and oncoprotein Tax. Viruses spread at the virological synapse (VS), a virus-induced specialized cell-cell contact, by polarized budding into synaptic clefts, and by cell surface transfer of viral biofilms (VBs). Since little is known about Tax’s role in formation of the VB, we asked which component of the VB is regulated by Tax and important for HTLV-1 transmission. Collagens are not only structural proteins of the extracellular matrix and basal membrane but also represent an important component of the VB. Here, we report that among the collagens known to be present in VBs, COL4 is specifically upregulated in the presence of HTLV-1 infection. Further, we found that transient expression of Tax is sufficient to induce *COL4A1* and *COL4A2* transcripts in Jurkat and CCRF-CEM T-cells, while robust induction of COL4 protein requires continuous Tax expression as shown in Tax-transformed T-cell lines. Repression of Tax led to a significant reduction of *COL4A1/A2* transcripts and COL4 protein. Mechanistically, luciferase-based promoter studies indicate that Tax activates the *COL4A2* and, to a less extent, the *COL4A1* promoter. Imaging showing partial co-localization of COL4 with the viral Gag protein in VBs at the VS and transfer of COL4 and Gag to target cells suggests a role of COL4 in VB formation. Strikingly, in chronically infected C91-PL cells, knockout of *COL4A2* impaired Gag transfer between infected T-cells and acceptor T-cells, while release of virus-like particles was unaffected. Taken together, we identified COL4 (COL4A1, COL4A2) as a component of the VB and a novel cellular target of Tax with COL4A2 appearing to impact virus transmission. Thus, this study is the first to provide a link between Tax’s activity and VB formation by hijacking COL4 protein functions.

## Introduction

Human T-cell leukemia virus type 1 (HTLV-1) is a highly oncogenic retrovirus causing Adult T-cell Leukemia/Lymphoma (ATLL) or inflammatory diseases in up to 10% of infected individuals (Tagaya and Gallo, 2017). Worldwide, at least 5–10 million people are infected with this yet neglected human retrovirus, however, it is estimated that there is a much higher number of unknown cases since statistics on HTLV-1 prevalence are lacking for several densely populated regions (Gessain and Cassar, 2012). HTLV-1 is highly endemic in Southwestern parts of Japan, Sub-Saharan Africa, South America, the Caribbean, and parts of the Middle East and Australo-Melanesia (Gessain and Cassar, 2012). In central Australia, up to 48% of certain ethnic communities in the socially disadvantaged Indigenous population are HTLV-1-infected (Einsiedel et al., 2016), which currently accounts for global worries (Martin et al., 2018). Since up to 90% of infected patients stay lifelong asymptomatic and blood donors are not screened for HTLV-1 infection in most countries, asymptomatic carriers are mainly unaware of their infection and may pass the infection to other people (Caswell et al., 2019). HTLV-1 is transmitted via cell-containing body fluids such as breast milk, blood products, semen, and via organ transplants (Pique and Jones, 2012; Gross and Thoma-Kress, 2016). Upon binding to its receptor, HTLV-1 infects its target cells, which are mainly CD4^+^ T-cells and to a less extent CD8^+^ T-cells, dendritic cells (DC), or monocytes (Macatonia et al., 1992; de Castro-Amarante et al., 2015; Melamed et al., 2015). Recent work suggests that infection of hematopoietic stem cells contributes to spread of HTLV-1 *in vivo* (Furuta et al., 2017).

Upon infection and reverse transcription, HTLV-1 integrates into the host cell genome and persists *in vivo* mainly in its provirus form (9.1 kb), which is flanked by long terminal repeats (LTR). In addition to structural proteins and enzymes common for retroviruses, HTLV-1 encodes regulatory (Tax, Rex) and accessory (p12/p8, p13, p30, HBZ) proteins (Currer et al., 2012). HTLV-1 replicates either by infecting new cells or by mitotic division and clonal proliferation of infected CD4^+^ T-cells. Cell-free transmission of HTLV-1 between T-cells is inefficient, free virions can hardly be detected in infected individuals and are poorly infectious for most cell types (Fan et al., 1992; Derse et al., 2001; Alais et al., 2015; Demontis et al., 2015). Efficient infection of CD4^+^ T-cells requires cell-cell contacts, and virus propagation from cell-to-cell depends on specific interactions between cellular and viral proteins. Two types of cell-cell contacts seem to be critical for HTLV-1 transmission: tight cell-cell contacts and cellular conduits (Igakura et al., 2003; Van Prooyen et al., 2010; Gross and Thoma-Kress, 2016). For transmission at tight cell-cell contacts, two non-exclusive mechanisms of virus transmission at the virological synapse (VS), a virus-induced specialized cell-cell contact, have been proposed, polarized budding of HTLV-1 into synaptic clefts (Igakura et al., 2003), and cell surface transfer of so-called viral biofilms (VBs) at the VS (Pais-Correia et al., 2010). In VBs, extracellular concentrated viral particles are embedded in a carbohydrate-rich structure that is induced and spatially reorganized by viral infection. In detail, viral assemblies are surrounded by cellular lectins (Galectin-3), heparan sulfate proteoglycans (Agrin), Tetherin (BST-2 or CD317), and components of the extracellular matrix like collagens of unknown composition (Pais-Correia et al., 2010). Further, monoclonal antibody screening revealed that the antigens CD4, CD150, CD70, CD80, and CD25 are concentrated in the VB and the latter three are inducible by Tax (Tarasevich et al., 2015). HTLV-1 transmission via VBs seems to constitute a major route of transmission *in vitro* since removal of biofilms severely impairs cell-to-cell transmission (Pais-Correia et al., 2010). Further, *in vitro* studies have shown that DC can be infected cell-free with high concentrations of isolated VBs, which then mediate efficient cell-cell contact-dependent infection of CD4^+^ T-cells (Alais et al., 2015). Moreover, recent work identified isolated viral biofilm-like structures as new viral structures activating innate immunity by triggering type I interferon (IFN) production of plasmacytoid DCs (Assil et al., 2019).

Among the HTLV-1-encoded proteins, the viral regulatory protein Tax is a crucial regulator of virus transmission. Tax is not only essential for HTLV-1 replication by transactivating the HTLV-1 *LTR* (*U3R*) promoter, but Tax is also a potent transactivator of cellular transcription, important for initiating oncogenic transformation, and crucial for viral spread (Chevalier et al., 2012; Currer et al., 2012). Tax regulates virus transmission by inducing and cooperating with intercellular adhesion molecule 1 (ICAM-1) and inducing polarization of the microtubule organizing center (MTOC), which leads to formation of the VS (Fukudome et al., 1992; Nejmeddine et al., 2005, 2009). Recent work suggests that next to Tax also HBZ contributes to HTLV-1-infectivity by upregulating ICAM-1 (Fazio et al., 2019). Use of single-cycle replication-dependent HTLV-1 reporter vectors revealed that Tax also enhances actin- and tubulin-dependent transmission of HTLV-1 virus-like particles (Mazurov et al., 2010). Cellular factors that are crucial for HTLV-1 infection are rather poorly described in the context of virus transmission. We and others could show that Tax interferes with a variety of cellular genes involved in virus transmission (Kress et al., 2011; Mazurov et al., 2012; Chevalier et al., 2014; Gross et al., 2016). We could show that Tax enhances HTLV-1 cell-to-cell transmission by inducing the actin-bundling protein Fascin (Kress et al., 2011; Gross et al., 2016), potentially by facilitating the recruitment of viral particles to budding sites. Although Tax had been shown to regulate several host cell factors playing a role in virus transmission (Pique and Jones, 2012; Gross and Thoma-Kress, 2016), little is known about Tax’s role in formation of the VB (Tarasevich et al., 2015). Here, we propose that Tax regulates a certain collagen which is part of the VB.

The collagen superfamily is composed of 29 different subtypes (COL I- XXIX, or COL1-29) with collagens representing the main structural proteins in various species and tissues. Collagens are important for structure maintenance, tensile stress resistance, cell adhesion, migration, cell–cell interactions, and chemotaxis (Leitinger, 2011). Collagen IV (COL4), a network-building collagen of the basal membrane, is not only a scaffold protein of the extracellular matrix, but it also binds to different integrin and non-integrin receptors to fulfill its various functions. The COL4 family consists of the six family members COL IV α1 (COL4A1) – COL IV α6 (COL4A6) (Khoshnoodi et al., 2008; Leitinger, 2011). Two COL4A1 (ca. 160 kDa each) and one COL4A2 polypeptide chains (ca. 167 kDa) form a tightly packed triple helix (COL4) by twisting around each other. COL4 consists of an N-terminal collagenous domain and a C-terminal non-collagenous domain (NC1) (Leitinger, 2011). *COL4A1* and *COL4A2* genes are located head-to-head on opposite strands and transcriptional regulation of *COL4A1* and *COL4A2* is controlled by a bi-directional promoter region and further regulatory elements located distantly (Poschl et al., 1988; Soininen et al., 1988). CD4^+^ T-lymphocytes, the main target cell type of HTLV-1 *in vivo*, do not express COL4. However, analysis of the VB in chronically HTLV-1-infected T-cell lines and primary T-cells from HTLV-1-infected patients using a pan-collagen antibody recognizing collagens 1-5 revealed that collagens are enriched in VBs (Pais-Correia et al., 2010).

Here, we report that among the collagens known to be present in VBs, COL4 is specifically upregulated in the presence of HTLV-1 infection and Tax is sufficient to induce *COL4A1* and *COL4A2* transcripts, while robust induction of COL4 protein requires continuous Tax expression. Imaging showing partial co-localization of COL4 with the viral Gag protein in VBs at the VS and transfer of COL4 and Gag to target cells suggests a role of COL4 in VB formation. Strikingly, in chronically infected C91-PL cells, repression of COL4 impaired Gag transfer between infected T-cells and acceptor T-cells, while release of virus-like particles was unaffected. Thus, this study is the first to provide a link between Tax’s activity, VB formation, and virus transmission by hijacking COL4 protein functions.

## Materials and Methods

### Cell Lines

The HTLV-1 *in vitro* transformed CD4^+^ T-cell lines C8166-45 (Salahuddin et al., 1983), MT-2 (Yoshida et al., 1982) and C91-PL (Ho et al., 1984), as well as the ATL-derived CD4^+^ T-cell line HuT-102 (Gazdar et al., 1980) and the CD4^+^ T-cell lines JPX9/JPX9M (Ohtani et al., 1989) carrying a cassette of Tax wildtype (JPX9) or of a mutated Tax (JPX9M) under control of a metallothioenin-sensitive promoter, were cultured in RPMI 1640 medium (GIBCO, Life Technologies, Darmstadt, Germany) containing 10% fetal calf serum (FCS; Sigma Aldrich, Darmstadt, Germany), L-glutamine (0.35 g/l) and penicillin/streptomycin (Pen/Strep; 0.12 g/l each). Induction of Tax or Tax mutant expression in JPX9/JPX9M cells was triggered by addition of 20 μM cadmium chloride (CdCl_2_) to the culture medium. The HTLV-1 *in vitro* transformed CD4^+^ T-cell line MS-9, containing only one single proviral genome (Shuh et al., 1999), as well as the Tax-transformed CD4^+^ T-cell lines Tesi (Schmitt et al., 1998), Tri (Grassmann et al., 1989) and TAXI-1 (Waldele et al., 2004), were kept in RPMI 1640 (40%) and Panserin 401 medium (40%; PAN-Biotech, Aidenbach, Germany), supplemented with 20% FCS, L-glutamine, Pen/Strep and 100 U/ml (MS-9), 40 U/ml (Tesi, Tri) or 20 U/ml (TAXI-1) interleukin-2 (IL-2; Roche Diagnostics, Mannheim, Germany). For repression of Tax protein in Tesi cells, 1 μg/ml tetracycline (Tet) was added to the culture medium for 10 days. The CD4^+^ T-cell lines Jurkat (Schneider et al., 1977), HuT-78 (Gazdar et al., 1980), CCRF-CEM (Foley et al., 1965), Molt-4 (Minowada et al., 1972), and the primary effusion lymphoma derived B-cell line JSC-1 (Cannon et al., 2000) were cultivated in RPMI 1640 (45%) and Panserin 401 medium (45%), supplemented with 10% FCS, L-glutamine and Pen/Strep. The Burkitt lymphoma derived B-cell lines Bjab (Menezes et al., 1975) and Raji (Pulvertaft, 1964), the Hodgkin lymphoma derived B-cell lines KM-H2 (Kamesaki et al., 1986), L-428 (Schaadt et al., 1979) and lymphoid cell line HDLM-2 (Drexler et al., 1986), as well as the primary effusion lymphoma derived B-cell line BC-3 (Arvanitakis et al., 1996) and the recombinant, Tio-expressing Herpesvirus saimiri (HVS) C488 transformed peripheral blood lymphocyte cell lines 1765 and 1766 (Albrecht et al., 2004) were cultured in RPMI 1640 (45%) and Panserin 401 medium (45%), supplemented with 10% FCS, L-glutamine and gentamycin (0.1 g/l). The StpC/Tip-expressing HVS C488 immortalized human cord blood lymphocyte cell line 1851/1 was kindly provided by B. Biesinger and J.C. Albrecht (Institute of Clinical and Molecular Virology, Erlangen, Germany). Cells were kept in RPMI 1640 (45%) and Panserin 401 medium (45%), supplemented with 10% FCS, L-glutamine, gentamycin and 10 U/ml IL-2. HEK-293T were cultured in DMEM (GIBCO, Life Technologies) containing 10% FCS, L-glutamine and Pen/Strep. In the case of C91-PL cells stably transduced with Clustered Regularly Interspaced Short Palindromic Repeats (CRISPR) constructs, 2 μg/ml puromycin was added to the culture medium.

### Primary Cells From Healthy Individuals

Peripheral blood mononuclear cells (PBMC) from healthy individuals were isolated from leukocyte cones derived from thrombocyte apheresis in the transfusion medicine section of the University Hospital Erlangen, which was approved by the Ethics Committee of the Medical Faculty of Friedrich-Alexander-Universität Erlangen-Nürnberg (Az. 220_16B; Erlangen, Germany). PBMC were isolated by density gradient centrifugation using Biocoll Separating Solution (Biochrom GmbH, Berlin, Germany). Isolated PBMC were subsequently cultivated with a density of 2^∗^10^6^ cells/ml in RPMI 1640 containing 10% FCS, L-glutamine and Pen/Strep. For stimulation of PBMC, cells were incubated in PBMC medium permanently containing 50 U/ml IL-2 and initially 2 μg/ml phytohemagglutinin (PHA) overnight.

### Patient Samples and Controls

Blood samples from HTLV-1 infected patients were obtained from the CHU of Martinique. Clinical collections of samples for research purpose are stored at the Center of Biological Resources of Martinique (CeRBiM). HTLV-1 asymptomatic carrier (AC) patients were recruited according to World Health Organization (WHO) criteria. AC had no neurologic or haematological symptoms. HAM/TSP patients were subgrouped according to their progression rate into slowly (TSP-L) or rapidly progressing patients (TSP-R). Rapid progressors are patients deteriorated more than three grades of a motor disability score within 2 years after initial examination (Olindo et al., 2006). According to the French Bioethics laws, the collection of samples has been declared to and approved by the ethics committee of the French Ministry of Research. Because the protocol is non-interventional, no informed consent was required, as stated by the French Public Health code and therefore the study was conducted anonymously. Non-infected CD4^+^ control T-cells were isolated from PBMC derived from buffy coats of healthy blood donors using the MACS negative depletion system (Miltenyi Biotec, Auburn, CA, United States). No contaminating CD8^+^ T cells, B-cells, monocytes, or natural killer cells were detected by flow cytometry. Purified CD4^+^ T cells were then cultured in RPMI 1640 medium (GIBCO, Life Technologies) supplemented with 10% FCS from Sigma Aldrich, 25 mM Hepes buffer, 2 mM L-glutamine, 1 mM sodium pyruvate and 50 μM 2-β-mercaptoethanol. CD4^+^ T cells (1^∗^10^6^/well) were activated in flat-bottomed 6-well plates pre-coated with the anti-CD3 mAb (10 μg/ml) in the presence of soluble anti-CD28 mAb (1 μg/ml). Cultures were harvested after 1 week for total RNA isolation.

### Microarray Analysis

Transcriptome analysis was carried out on the Affymetrix HGU133plus 2.0 platform (Affymetrix, Santa Clara, CA, United States) in biological replicates as described before (Pichler et al., 2008; Kress et al., 2010). Briefly, the transcriptome of Tax-expressing Tesi cells was compared to that of Tesi/Tet cells, where Tax expression was repressed. Further, the HTLV-1 *in vitro*-transformed cell line (MT-2) was compared to postmitotic CD4^+^ T lymphocytes. The microarray data have been deposited in NCBI’s Gene Expression Omnibus (Edgar et al., 2002) and are accessible at www.ncbi.nlm.nih.gov/geo and GEO Series accession numbers are GSE10508 and GSE17718.

### Quantitative Real-Time RT-PCR (qPCR) and RT-PCR

Total cellular RNA from transfected cells or untransfected cell lines was isolated (*NucleoSpin*^®^ *RNA*, Macherey Nagel, Düren, Germany) and reversely transcribed to cDNA by applying *random hexamer primers* and *Superscript*^TM^ *II Reverse Transcriptase* (both Thermo Fisher Scientific, Waltham, MA, United States) according to the manufacturers’ instructions. Quantitative real-time RT-PCR (qPCR) was performed using 200 ng of cDNA and *SensiMix*^TM^ *II Probe Kit* (Bioline GmbH, Luckenwalde, Germany) or *TaqMan*^®^ *Universal PCR Master Mix* in an *ABI Prism 7500 Sequence Analyzer* (both Applied Biosystems, Foster City, CA, United States) according to the manufacturers’ instructions. Primer sequences and FAM (6-carboxyfluorescein)/TAMRA (tetramethylrhodamine)-labeled probes for detection of β*-actin* (ACTB) and *Tax* transcripts have been described before (Pichler et al., 2008). *COL4A1* and *COL4A2* transcripts were detected using *TaqMan Gene Expression Assays* Hs00266237_m1 (COL4A1) and Hs01098858_m1 (COL4A2; both Applied Biosystems). Transcript expression levels were computed from standard curves generated by pJET1.2/blunt plasmids (Fermentas, St.-Leon Roth, Germany) bearing respective target sequences and the mean of technical triplicates was calculated for all samples. Every experiment was independently performed at least three times and relative copy numbers (rcn) were calculated by normalization of respective transcript levels on those of β*-actin*. Total RNA from HTLV-1-infected patients and uninfected controls was prepared from whole cells using Trizol (Invitrogen, Thermo Fisher Scientific) as previously described (Terol et al., 2017). After reverse transcription (RT) using 5x All-In-One RT MasterMix (ABM, Vancouver, BC, Canada), the abundance of transcripts was assessed by qPCR analysis using the SYBR green PCR master mix (Roche Diagnostics) and gene-specific primer sets. Data were analyzed using LightCycler^®^ 480 Software (Roche Diagnostics). Primers for *COL4A1* (qHsaCID0010223) and *COL4A2* (qHsaCED0044576) and the housekeeping gene *HPRT-1* (qHsaCID0016375) were from BioRad (Hercules, CA, United States). For qualitative analysis of mRNA expression, 500 ng of cDNA were subjected to RT-PCR using dNTPs (250 μM each) and *DreamTaq DNA polymerase* (2 U, both Thermo Fisher Scientific). Primer (0.6 μM each) sequences were as follows: Tax-RT-fwd 5′-CAGCCCAC TTCCCAGGGTTTGGAC-3′, Tax-RT-rev 5′-GTGTGAGAGT AGAAATGAGGGGT-3′, ACTB-RT-fwd 5′-CGGGAAATCGTG CGTGACAT-3′, ACTB-RT-rev 5′-GAACTTTGGGGGATGCT CGC-3′.

### Western Blot

Cell lines or transfected cells were resuspended in lysis buffer [150 mM NaCl, 10 mM Tris/HCl (pH 7.0), 10 mM EDTA, 1% Triton^TM^ X-100, 2 mM DTT and protease inhibitors leupeptin, aprotinin (20 μg/ml each) and 1 mM phenylmethylsulfonyl fluoride (PMSF)] and subjected to repeated *freeze-and-thaw* cycles between −196°C (liquid nitrogen) and 30°C. In the case of Tax protein detection, lysates were additionally sonicated three times for 20 s. Equal amounts of proteins (between 30 and 50 μg) were denatured for 5 min at 95°C in sodium dodecyl sulfate (SDS) loading dye (10 mM Tris/HCl (pH 6.8), 10% glycerin, 2% SDS, 0.1% bromophenol blue, 5% β-mercaptoethanol). Samples were subjected to SDS-PAGE and immunoblot using nitrocellulose transfer membranes (*Whatmann*^®^, *Protran*^®^, Whatmann GmbH, Dassel, Germany) or *Immobilon*^®^*-FL* PVDF transfer membranes (Merck Millipore, Billerica, MA, United States) using standard protocols. Proteins were detected with the following primary antibodies: rabbit polyclonal anti-COL4 (ab6586, Abcam), mouse anti-Tax (derived from the hybridoma cell line 168B17-46-34, provided by B. Langton through the AIDS Research and Reference Reagent Program, Division of AIDS, NIAID, NIH, Langton et al., 1988), rabbit polyclonal anti-Tio serum (a kind gift of Brigitte Biesinger and Jens Albrecht, Albrecht et al., 1999), mouse monoclonal anti-HTLV-1 gag p19 (TP-7, ZeptoMetrix Corporation), mouse monoclonal anti-GFP (GSN24, Sigma), mouse monoclonal anti-Hsp90 α/β (F-8, Santa Cruz Biotechnology), mouse monoclonal anti-α-Tubulin (T9026, Sigma), mouse monoclonal anti-ß-actin (AC-15, Sigma) and mouse monoclonal anti-GAPDH (3B1E9, GenScript). For detection of proteins blotted on nitrocellulose membranes, secondary antibodies anti-mouse or anti-rabbit conjugated with horseradish peroxidase (HRP; GE Healthcare, Little Chalfont, United Kingdom) were employed. For detection of PVDF-transferred proteins, secondary antibodies anti-mouse or anti-rabbit Alexa Fluor^®^ 647 (Life Technologies GmbH) were applied. Peroxidase activity was assessed by enhanced chemiluminescence using a CCD camera (Fujifilm LAS-1000 Intelligent Dark Box; Fujifilm). Fluorescence signals were detected using the Advanced Fluorescence Imager camera (ChemoStar, Intas Science Imaging GmbH, Göttingen, Germany).

### Confocal Laser Scanning Microscopy

#### Detection of COL4 Protein Expression

For detection of COL4 protein expression, 1.8^∗^10^5^ MT-2, HuT-102, C91-PL, C8166-45, or Jurkat T-cells were resuspended in 30 μl PBS and spotted on epoxy-resin coated glass slides (medco Diagnostika GmbH, Hengersberg, Germany) or on poly-L-lysine coated coverslips by gentle desiccation. Cells were fixed in 2% paraformaldehyde (PFA; 1 h, 25°C) and washed five times with PBS/0.1% Tween^®^ 20. Permeabilization was performed with PBS/0.2% Triton^TM^ X-100 (20 min, 4°C) where indicated, or cells were left unpermeabilized by incubation in mere PBS (20 min, 4°C). Cells were washed twice and incubated with PBS/5% FCS/1% bovine serum albumin (BSA; 1 h, 25°C). Both primary antibodies, rabbit polyclonal anti-COL4 (ab6586, Abcam) and mouse monoclonal anti-CD98 (ab2528, Abcam), were applied together in blocking solution (45 min, 37°C). Cells were washed three times and incubated with secondary antibodies anti-mouse Alexa Fluor^®^ 488 and anti-rabbit Alexa Fluor^®^ 647 (both Life Technologies GmbH) in blocking solution (45 min, 37°C) one after the other with three washing steps in between. Finally, samples were covered with *ProLong*^TM^ *Gold Antifade Mountant with DAPI* (Life Technologies GmbH) according to the manufacturer’s instructions. Images were acquired using a *Leica TCS SP5* confocal laser scanning microscope equipped with a 63 × 1.4 HCX PL APO CS oil immersion objective lens (Leica Microsystems GmbH, Wetzlar, Germany). Images were analyzed using *LAS AF software* (Leica) and *Adobe Photoshop CS5* (Adobe Systems, San Jose, CA, United States).

#### Co-culture Assays Between HTLV-1 Infected MS-9 Cells and Uninfected Jurkat T-cells

HTLV-1 negative Jurkat acceptor cells were prestained with the live cell dye *CellTracker*^TM^ *Blue 7-Amino-4-Chlormethylcumarin* (CMAC; Thermo Fisher Scientific; 20 μM, 45 min, 37°C). After five washing steps in serum-free medium, prestained Jurkat T-cells and HTLV-1 positive MS-9 donor cells were co-cultured at a ratio of 1:1 on poly-L-lysine-coated coverslips (20 or 50 min, 37°C) and fixed with 2% PFA (1 h, 25°C). Cells were permeabilized and stained as described in Detection of COL4 Protein Expression. Primary antibodies mouse monoclonal anti-HTLV-1 gag p19 (TP-7, ZeptoMetrix Corporation) and rabbit polyclonal anti-COL4 in blocking solution (45 min, 37°C) were used. Secondary antibody staining was performed as described in “Detection of COL4 Protein Expression.” Mounting of cells was performed with *ProLong*^TM^ *Gold Antifade Mountant without DAPI* (Life Technologies GmbH). After acquisition (see Detection of COL4 Protein Expression), images were analyzed and signal intensities were quantified using *LAS AF software* (Leica) and *Adobe Photoshop CS5* (Adobe Systems). For quantitative evaluation, manual counting of cells was performed, analyzing in total 1524 cells of 83 optical fields. Transfer of Gag and/or COL4 protein from MS-9 donor cells to Jurkat acceptor cells was determined and normalized on the number of Gag or COL4 positive MS-9 cells and on the ratio of Jurkat acceptor to MS-9 donor cells.

### Plasmids and Transfection

#### Plasmids

The following plasmids were used: the Tax expression vectors pEFneo-Tax1 (Shoji et al., 2009) and pc-Tax (Rimsky et al., 1988); pEFneo and pcDNA3.1 (Life Technologies; controls); the Rex-GFP expression plasmid pCMV-Rex1-GFP (kind gift from Donna M. D’Agostino, University of Padova); the luciferase-reporter control vector pGL3-Basic (Promega, Mannheim, Germany), luciferase-reporter vectors harboring the *U3R* sequence of the HTLV-1 LTR pGL3-U3R-Luc (U3R-Luc; Mann et al., 2014) or sequences of the *COL4A1* or *COL4A2* promoter pGL3-COL4A1-Luc (COL4A1-Luc) and pGL3-COL4A2-Luc (COL4A2-Luc) (Turner et al., 2015); the HTLV-1 packaging vector pCMV-HT1MΔXho (HT1MΔEnv) harboring parts of the HTLV-1 proviral genome lacking the envelope protein (Derse et al., 2001).

#### Transfection

For COL4 induction, 10^∗^10^6^ Jurkat or CCRF-CEM T-cells were transfected with 100 μg Tax expression plasmid or the empty vector control. Cells were separated before harvest to generate RNA and Western Blot lysates. For luciferase reporter assays, 5^∗^10^6^ Jurkat cells were transfected with 30 μg Tax expression plasmid and 20 μg of the respective reporter vector. Jurkat and CCRF-CEM T-cells were electroporated using the *Gene Pulser X Electroporation System* (BioRad) at 290 V and 1500 μF.

### Generation of Stable Knockout C91-PL Cells

For knockout of *COL4A1* and *COL4A2* by CRISPR technology, the lentiCRISPRv2 vector system was employed [Sanjana et al., 2014; kind gift from Feng Zhang (Addgene plasmid #52961)]. The guide RNA sequence of pCAS-Scramble (OriGene; GCACTACCAGAGCTAACTCA), a non-specific RNA sequence, served as negative control. Guide-RNA sequences targeting *COL4A1* and *COL4A2* were designed with the CRISPR gRNA Design tool (DNA2.0, Atum, Newark, CA, United States) and were for *COL4A1*: CCAGGAGGTCCCGGTTCACC (COL4A1_1) and GTGCTCCTCGTGGAGCAGAA (COL4A1_2) and for *COL4A2*: CCTGGAGACGCCGGCTTACC (COL4A2_1) and GAAAGTCGCTTACCGCCGTA (COL4A2_2). Oligos were inserted into lentiCRISPRv2 vector replacing the 2 kb filler sequence by Bsm*BI* restriction enzyme via standard cloning procedures. The resulting vectors were designated lentiCRISPRv2-scramble-guide (scramble), lentiCRISPRv2-COL4A1-guide1 (COL4A1_1), lentiCRISPRv2-COL4A1-guide2 (COL4A1_2), lentiCRISPRv2-COL4A2-guide1 (COL4A2_1) and lentiCRISPRv2-COL4A2-guide2 (COL4A2_2). For lentiviral production, 5^∗^10^6^ 293T cells were seeded in 10 cm dishes and 24 h later, cells were transfected with *GeneJuice*^®^ transfection reagent (Merck Millipore, Darmstadt, Germany) according to the manufacturer’s protocol using a total amount of 15 μg DNA: 6 μg CRISPR vector, 6 μg HIV-1 gag-pol expression vector psPAX2 and 3 μg VSV-G expression vector pMD2.G (kind gifts from Didier Trono, Addgene plasmids #12260 and #12259, respectively). At 72 h after transfection, lentivirus-containing supernatants were concentrated (4000 g, 15 min) using Amicon^®^ Ultra^®^-15 Centrifugal Filters (Ultracel-100K; Merck Millipore). 1^∗^10^6^ C91-PL cells were spin-infected with 1.5 ml concentrated virus (150 g, 2 h, 32*°*C) and adjusted to 2^∗^10^5^ C91-PL cells/ml. CRISPR-vectors COL4A1_1 and COL4A1_2 or COL4A2_1 and COL4A2_2 were transduced as pools to generate a COL4A1 or a COL4A2 knockout. Initial selection was performed 72 h after transduction by addition of 2 μg/ml puromycin to the cell culture medium.

#### Luciferase Reporter Assays

Jurkat T-cells were transfected, as described above, in quadruplicates. One sample was subjected to western blot analysis as described above. The remaining triplicate samples were processed for luciferase reporter assays determining firefly luciferase activity as described before (Mann et al., 2014). Relative light units (RLU) obtained from U3-Luc, COL4A1-Luc or COL4A2-Luc were normalized on protein content and on the respective values derived from co-transfection with the pGL3-Basic negative control vector, which represents background activity.

### Gag p19 ELISA

0.5^∗^10^6^ stably transduced C91-PL cells were seeded in 24 well plates and incubated for 48 h. Supernatants were subsequently sterile filtrated by passing through a 0.45 μm filter and virus release was determined using gag p19 ELISA according to the manufacturer’s instruction (ZeptoMetrix Corporation, Buffalo, NY, United States). Values were obtained using Softmax Pro Version 5.3 software (MDS Analytical Technologies, Sunnyvale, CA, United States). Five independent experiments, each performed in duplicate, were performed.

### Flow Cytometry

1^∗^10^6^ C91-PL-scramble, C91-PL-CRISPR-COL4A1, or C91-PL-CRISPR-COL4A2 cells were co-cultured with 1^∗^10^6^ Jurkat T-cells for 1 h at 37°C. The latter have been prestained with CMAC as described earlier (Donhauser et al., 2018). Co-cultures of Jurkat T-cells with C91-PL cells treated with cytochalasin D (5 μM, 24 h) or the solvent control DMSO served as controls. Cells were stained using mouse monoclonal antibodies anti-gag p19 (ZeptoMetrix Corporation) and anti-mouse AlexaFluor^®^ 647-conjugated secondary antibodies (Life Technologies GmbH) as described earlier (Gross et al., 2016). Cells were discriminated by CMAC-staining (Jurkat: CMAC-positive; C91-PL: CMAC-negative) and their different size (FSC/SSC). The percentage of Gag-positive cells within CMAC-positive cells (Jurkat T-cells) was examined to measure Gag transfer from C91-PL to Jurkat T-cells.

## Results

### COL4 Is Specifically Upregulated in HTLV-1-Infected T-cells

**FIGURE 1:**
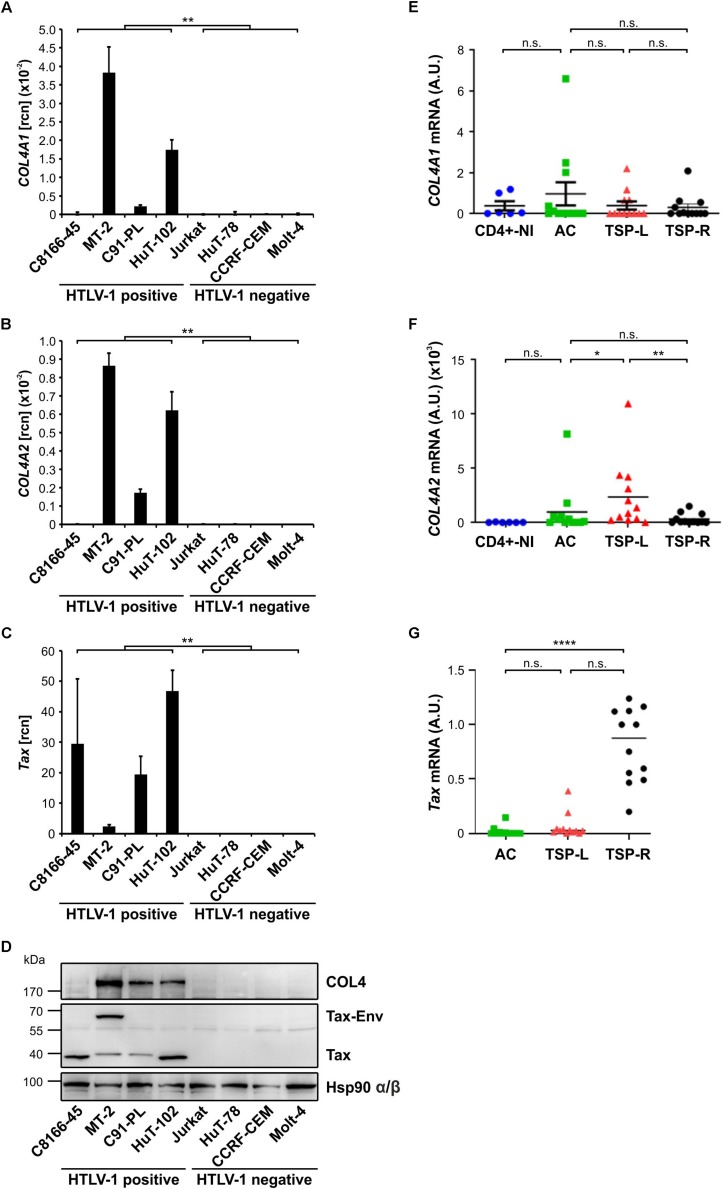
COL4 is specifically upregulated in HTLV-1 positive T-cell lines as well as *COL4A2* in *ex vivo* HAM/TSP patient samples. **(A–D)** Analysis of COL4 expression in HTLV-1 positive (C8166-45, MT-2, C91-PL, HuT-102) and HTLV-1 negative T-cell lines (Jurkat, HuT-78, CCRF-CEM, Molt-4). **(A–C)** mRNA of **(A)** *COL4A1*, **(B)** *COL4A2*, and **(C)** *Tax* was quantified by qPCR in the respective HTLV-1 positive and negative T-cell lines. The mean relative copy numbers (rcn), normalized on *ACTB*, of three individual experiments ± SE are depicted. Values were compared using two-tailed Mann–Whitney *U* test (^∗∗^*p* < 0.01). **(D)** COL4 protein was detected in HTLV-1 positive and negative T-cell lines by means of Western Blot. Staining of Tax and Hsp90 α/β served as expression or loading control, respectively. **(E)** *COL4A1*, **(F)** *COL4A2*, and **(G)** *Tax* transcripts were quantified by qPCR comparing mRNA isolated from patient samples of HTLV-1 asymptomatic carriers (AC), slowly progressing HAM/TSP patients (TSP-L), rapidly progressing HAM/TSP patients (TSP-R), and uninfected controls (CD4^+^-NI). Statistical analysis was performed applying two-tailed Mann–Whitney *U* test (^∗^*p* < 0.05; ^∗∗^*p* < 0.01; ^****^p < 0.0001; n.s., not significant).

Viral biofilms (VBs) depict, next to formation of the virological synapse (VS), a fundamental role in HTLV-1 transmission via close cell-to-cell contacts (Pais-Correia et al., 2010). Several cellular factors have been identified to account for the formation of VB (Pais-Correia et al., 2010), but only little is known about the involvement of HTLV-1/Tax in regulating expression of these cellular components of the VB (Tarasevich et al., 2015). Thus, we re-evaluated microarray data we had performed earlier (Pichler et al., 2008; Kress et al., 2010) and analyzed the expression of the initially identified components of the VB (Pais-Correia et al., 2010) on their ability to be induced by HTLV-1/Tax. For this purpose, we compared the transcriptome of HTLV-1 positive (MT-2) or Tax-positive (Tesi) T-cells to respective HTLV-1 negative (postmitotic CD4^+^) or Tax-negative (Tesi/Tet) T-cells. Briefly, Tesi cells had been established by transforming human cord blood lymphocytes with a tetracycline-repressible *Tax* gene using a rhadinoviral vector (Schmitt et al., 1998). Addition of tetracycline (Tet; 1 μg/ml, 10 days) to the culture medium leads to repression of Tax (Tesi/Tet) (Pichler et al., 2008; Kress et al., 2010). We could show that Agrin and Tetherin (BST2, bone marrow stromal antigen 2; CD317 antigen) are present in all cell culture systems independent of HTLV-1 infection or Tax-transformation (Table 1; see all probe sets in Supplementary Table 1). Fucosyltransferase 4 (FUT4), which is next to Fucosyltransferase 9 (FUT9) able to synthesize the carbohydrate sialyl LewisX (3-fucosyl-N-acetyl-lactosamine) (Nakayama et al., 2001) enriched in VBs (Pais-Correia et al., 2010), showed 2–3 fold elevated transcript levels in the presence of Tax only (Tesi vs. Tesi/Tet, MT-2 vs. CD4^+^, Table 1). Contrary, *FUT9* was absent, suggesting that FUT4 is responsible for synthesis of sialyl LewisX in HTLV-1/Tax-positive cells. On the other hand, mRNA levels of *Galectin-3* (*LGALS3*) were present, but only slightly upregulated in one cell culture model (MT-2 vs. CD4^+^, Table 1). Interestingly, throughout the group of Collagens type 1 to 5, only the heterotrimeric couple of COL4A1 and COL4A2 seemed to show elevated transcript levels (Table 1). Although also *COL1A1* seemed to be upregulated in the presence of HTLV-1/Tax, *COL1A2*, the matching partner of COL1A1 to form the COL1A1-A1-A2 heterotrimer, could not be detected in any cellular system we tested. Therefore, we further focused on COL4A1 and COL4A2 only. Confirming microarray data by quantitative PCR (qPCR), in the HTLV-1 positive T-cell lines MT-2, C91-PL and HuT-102 detectable amounts of *COL4A1* and *COL4A2* as well as *Tax* were present (Figures 1A–C). An exception is the cell line C8166-45 which expresses *Tax* but not *COL4A1* and *COL4A2* (Figures 1A–C). This T-cell line is HTLV-1 positive yet Rex-deficient and impaired for expression of the structural proteins Gag and Env and therefore does not produce infectious viral particles (Bhat et al., 1993), which may serve as an explanation for the lack of *COL4A1* and *COL4A2* transcripts. As expected, all HTLV-1 negative T-cell lines tested (Jurkat, HuT-78, CCRF-CEM, Molt-4) proved absence of *COL4A1*, *COL4A2* and *Tax* (Figures 1A–C). Tax protein expression in all HTLV-1 positive T-cells could be observed, with the typical additional Tax-Env fusion protein in MT-2 cells (Figure 1D). Corresponding to the presence or absence of *COL4A1* and *COL4A2* transcripts COL4 protein was detected on Western Blot level (Figure 1D). Since the microarray (Table 1) pointed out that all other members of the Collagen 4 family (COL4A3-COL4A6) showed absence of mRNA, we assumed that the Western Blot signal generated with the antibody targeting COL4 is specific for COL4A1 and COL4A2. Notably, only very little copy numbers of *COL4A1* and *COL4A2*, as low as 1^∗^10^–3^, are sufficient to induce robust COL4 protein expression, indicating great stability of this extracellular matrix protein. To prove uniqueness of COL4 upregulation by HTLV-1 in cellular transformation and tumor formation, we compared various tumor-derived B-cell lines on the presence of COL4 protein. B-cell lines from Burkitt lymphoma (BL; Bjab, Raji), Primary Effusion lymphoma (PEL; JSC-1, BC-3; Kaposi’s sarcoma-associated herpesvirus-positive) as well as Hodgkin lymphoma (HL; KM-H2, L428, HDLM-2) consistently lacked COL4 protein in comparison to the HTLV-1 positive T-cell line MT-2 (Supplementary Figure 1). Hence, upregulation of COL4 is not a general hallmark of cellular transformation *per se* but is specific for HTLV-1 oncogenesis. Moving from cell culture into human, we analyzed *ex vivo* samples from HAM/TSP patients in comparison to non-infected CD4^+^ T-cells. No differences of *COL4A1* mRNA could be observed in CD8^+^-depleted PBMC between non-infected donors (CD4^+^-NI), asymptomatic carriers (AC) and slowly (TSP-L) or rapidly progressing patients (TSP-R) (Figure 1E). Slowly progressing HAM/TSP patients showed statistically significant elevated transcript levels of *COL4A2* compared to non-infected donors (CD4^+^-NI), asymptomatic carriers and rapidly progressing patients (Figure 1F). Although expression of *Tax* did not correlate with *COL4A2* since TSP-R but not TSP-L express high level of *Tax* (Figure 1G), we cannot exclude that induction of COL4 by the virus might indeed affect and modulate onset of HTLV-1 associated diseases.

**TABLE 1:** Transcriptional expression of components of the viral biofilm.

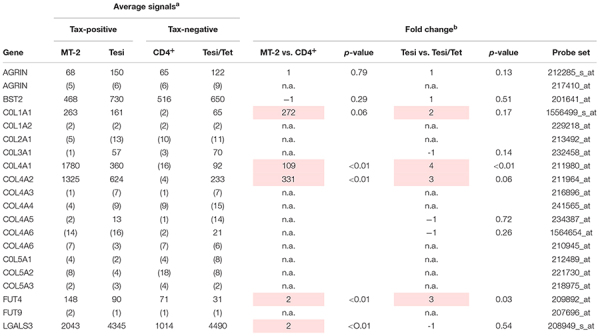

*^*a*^Average signals were rounded to integers; values in brackets were deemed absent calls. ^*b*^Values depicting the fold change of transcript expression were rounded to integers; *p*-values were rounded on two decimals; vs., versus; n.a., not applicable; red shaded, fold change ≥ 2.*

### COL4 Protein Localizes to Extra- and Intracellular Compartments in HTLV-1 Positive T-cells

**FIGURE 2:**
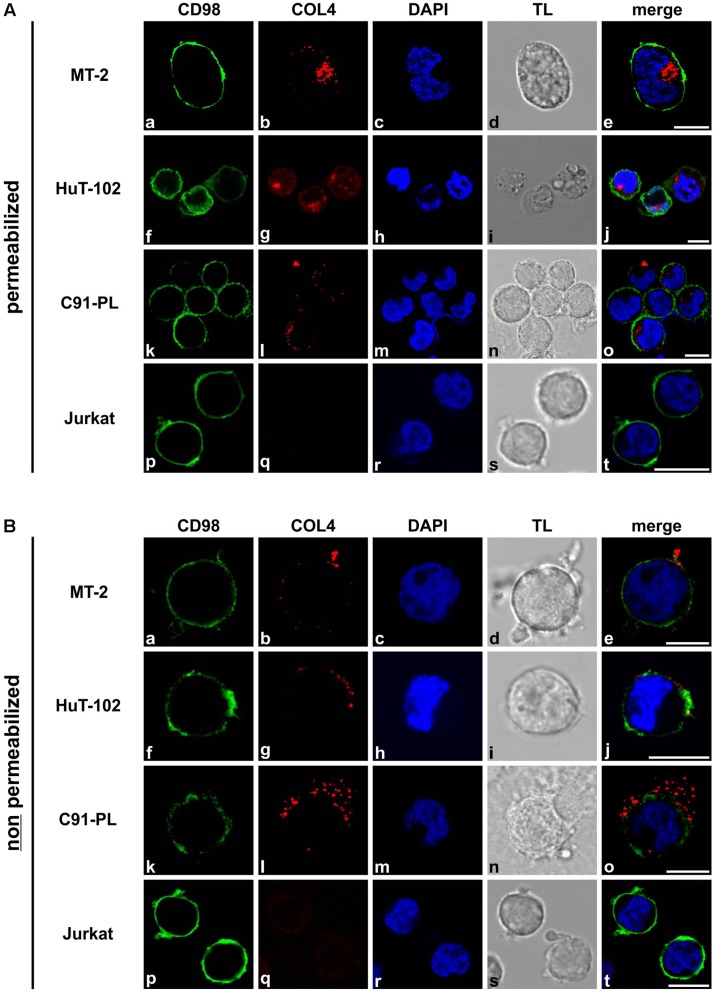
COL4 protein localizes to extra- and intracellular compartments in HTLV-1 positive T-cells. **(A,B)** Immunofluorescence staining was performed in the HTLV-1 positive T-cell lines MT-2, HuT-102 and C91-PL and in the HTLV-1 negative T-cell line Jurkat. Cells were spotted on epoxy-resin coated glass slides. The plasma membrane was visualized in green by using a mouse primary antibody against CD98, together with the secondary antibody anti-mouse Alexa Fluor^®^ 488. COL4 protein was depicted in red, employing a COL4 targeting rabbit primary antibody that was recognized by the secondary antibody anti-rabbit Alexa Fluor^®^ 647. DAPI staining of nuclei (blue) and transmitted light (TL) served as controls. Cells in **(Aa–t)** were permeabilized before antibody treatment, cells in **(Ba–t)** were left without permeabilization showing extracellular COL4 protein only. The scale bars represent 10 μm.

Collagens are proteins of the extracellular matrix (ECM) that are secreted upon translation and could be shown to contribute to formation of the VB (Schnoor et al., 2008; Pais-Correia et al., 2010). However, since antibodies recognizing collagens 1–5 were used in previous work (Pais-Correia et al., 2010), the exact subcellular localization of COL4 protein remained unclear. Therefore, we performed confocal microscopy comparing permeabilized and non-permeabilized HTLV-1 positive T-cells that had been spotted on epoxy-resin coated glass slides. Specifically, we costained COL4 protein with the same antibodies as used for western blot analysis (Figure 2, red) together with the plasma membrane marker CD98 (Figure 2, green), and with the DNA-tracer DAPI (Figure 2, blue) to be able to discriminate between extra- and intracellular portions of COL4 protein. Upon permeabilization, the HTLV-1 positive T-cell lines MT-2, HuT-102 and C91-PL proved to exhibit strong intracellular signals for COL4 protein whereas the HTLV-1 negative T-cell line Jurkat showed no COL4 protein (Figures 2Ab,g,l,q). In T-cells that have not been permeabilized, staining of COL4 protein could as well be detected in all HTLV-1 positive but not in Jurkat T-cells (Figures 2Bb,g,l,q). However, taking into account the signal of the plasma membrane marker CD98 (Figures 2Ba,f,k) and that cells had not been permeabilized, COL4 protein localized only to extracellular compartments of HTLV-1-infected cells in this setting (Figures 2Be,j,o). Of note, permeabilization of the cells may have negative impact on the detection of extracellular COL4 by destruction of collagenous structures since extracellular COL4 was nearly undetectable upon permeabilization (Figures 2Ab,g,l) in this setting. Similar results were obtained repeating the experiment staining HTLV-1 positive T-cells for Collagens 1 to 5, confirming findings obtained from Pais-Correia and colleagues (Supplementary Figure 2; Pais-Correia et al., 2010). Manual counting of cells confirmed that the number of COL4 or COL1-5 positive T-cells comparing permeabilized and non-permeabilized cells only slightly differed throughout the different cell types. However, upon permeabilization, the proportion of strong COL4 and COL1-5 signals vastly increased amongst all HTLV-1 positive T-cells (data not shown). In summary, COL4 protein localizes to both, intra- and extracellular, compartments in HTLV-1 positive cells.

### Transient Expression of Tax Is Sufficient to Induce *COL4A1* and *COL4A2* Transcripts but Not COL4 Protein

**FIGURE 3:**
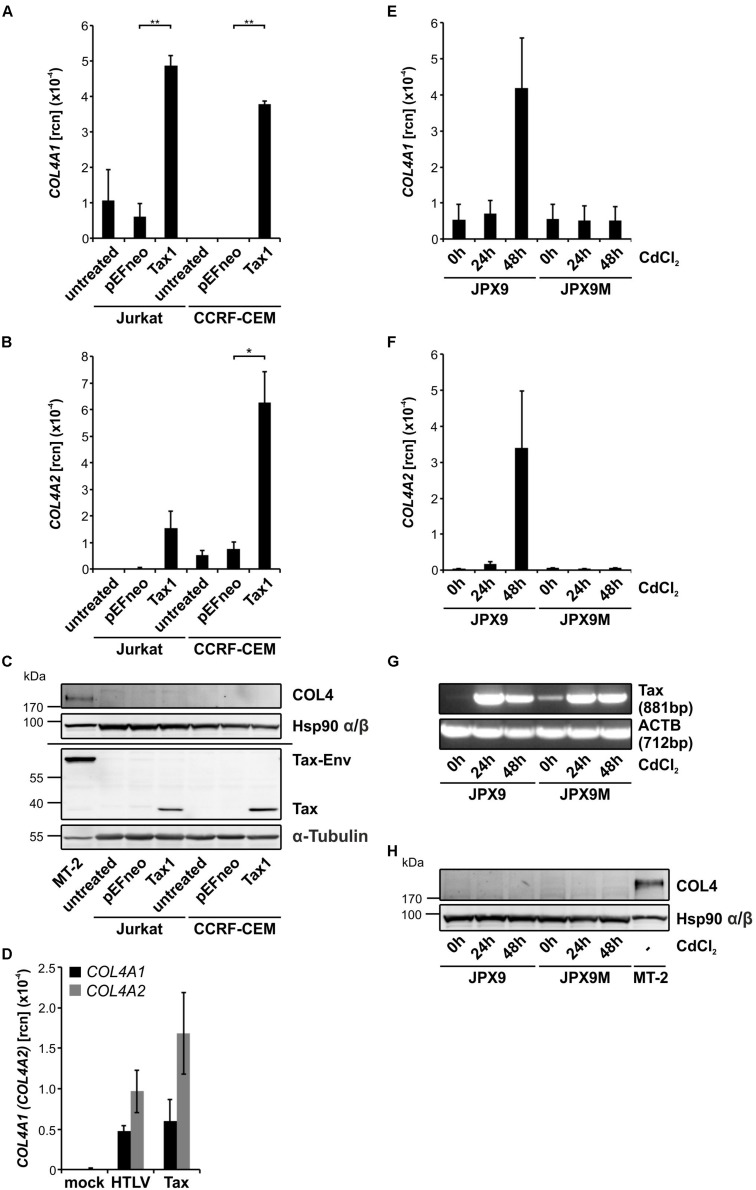
Transient expression of Tax is sufficient to induce *COL4A1* and *COL4A2* transcripts, but not COL4 protein. **(A)** *COL4A1* and **(B)** *COL4A2* transcript levels were quantified by qPCR upon transient expression of 100 μg Tax expression plasmid (pEFneo-Tax1) or empty vector (pEFneo) in 10^∗^10^6^ Jurkat and CCRF-CEM T-cells. The mean relative copy numbers (rcn), normalized on *ACTB*, of three independent experiments ± SE are depicted. Student’s *t*-test was conducted for statistical analysis (^∗^*p* < 0.05; ^∗∗^*p* < 0.01). **(C)** Immunoblotting for COL4 protein was performed in mock- (pEFneo) and Tax-transfected T-cell lines Jurkat and CCRF-CEM (100 μg DNA, 10^∗^10^6^ cells) as well as for MT-2 cells as positive control. Tax and GAPDH or α-Tubulin staining were carried out as controls. **(D)** *COL4A1* and *COL4A2* transcript levels were quantified by qPCR upon transient expression of 50 μg Tax expression plasmid (pcTax), 17.5 μg of the HTLV-1 packaging plasmid pCMV-HT1-ΔEnv (HTLV) supplemented with 32.5 μg empty vector (pcDNA), or 50 μg empty vector (pcDNA) alone in 5^∗^10^6^ Jurkat cells. The mean relative copy numbers (rcn) ± SE, normalized on *ACTB*, of one representative experiment is depicted. **(E,F)** mRNA of **(E)** *COL4A1* and **(F)** *COL4A2* transcripts was measured by qPCR in the Tax-inducible T-cell lines JPX9 and JPX9M at the indicated time points after induction of Tax wildtype protein (JPX9) or Tax mutant protein (JPX9M) by addition of 20 μM CdCl_2_. The mean relative copy numbers (rcn), normalized on *ACTB*, of three independent experiments ± SE are depicted. **(G)** For qualitative analysis, *Tax* and *ACTB* transcripts were amplified by RT-PCR from JPX9 and JPX9M cell lysates at the indicated time points after induction of Tax wildtype (JPX9) or Tax mutant protein (JPX9M). **(H)** Western Blot analysis was carried out staining COL4 protein as well as Hsp90 α/β as loading control at the indicated time points after induction of Tax wildtype (JPX9) or Tax mutant (JPX9M) protein expression. MT-2 cells served as positive control for COL4 protein expression.

The HTLV-1 oncoprotein Tax interferes with several cellular genes in the context of viral transmission (Nejmeddine et al., 2005; Chevalier et al., 2014; Gross et al., 2016; Gross and Thoma-Kress, 2016). Therefore, we asked whether Tax protein is responsible for induction of COL4. We transiently expressed Tax protein in the two T-cell lines Jurkat and CCRF-CEM by electroporation and performed qPCR and western blot 48 h after transfection. In the presence of Tax, *COL4A1* as well as *COL4A2* transcripts are indeed significantly elevated (Figures 3A,B). However, in spite of robust Tax protein expression, no COL4 protein could be detected next to the MT-2 control stain on Western Blot level (Figure 3C). Although we found that Tax alone is sufficient to upregulate *COL4A1* and *COL4A2* transcripts, we asked whether the extent of this upregulation could be different in the context of the HTLV-1 genome. However, comparable transcript levels of *COL4A1* and *COL4A2* were induced by either the HTLV-1 packaging plasmid pCMV-HT1-ΔEnv carrying parts of the HTLV-1 genome except Env and parts of the 5′LTR, or by Tax expression plasmids in Jurkat T-cells (Figure 3D). Yet, it cannot be excluded that another viral protein may affect COL4 transcript and protein expression. This is supported by the notion that the Rex-deficient cell line C8166-45 showed expression of Tax, but not of COL4 (Figures 1A–D). Our current findings argue against a role of Rex in regulating COL4 expression since co-expression of Rex-GFP and Tax did not further enhance *COL4A1* and *COL4A2* transcript levels (Supplementary Figure 3A), nor did co-expression of Rex-GFP induce COL4 protein (Supplementary Figure 3B). To confirm results obtained from transient transfection systems, we moved to JPX9/JPX9M, Tax-inducible T-cells which carry a Tax transgene controlled by a cadmium chloride (CdCl_2_)-inducible promoter (Ohtani et al., 1989). As corresponding control, JPX9M cells were employed, a T-cell line being able to induce a functionally inactive Tax mutant protein with a premature stop codon. At 0, 24, and 48 h after induction of Tax expression JPX9 and JPX9M cells were analyzed for expression of COL4 (Figures 3E–H). Confirming results obtained from Jurkat and CCRF-CEM T-cells, expression of Tax (Figure 3G) led to a time-dependent induction of *COL4A1* and *COL4A2* in JPX9 cells, reaching similar copy numbers as after transient transfection 48 h after addition of CdCl_2_ (Figures 3E,F). This effect was likely due to presence of Tax protein as expression of a Tax mutant variant in JPX9M T-cells was not sufficient to induce *COL4A1* or *COL4A2* (Figures 3E,F). However, in spite of *COL4A1* and *COL4A2* transcript induction by Tax, no COL4 protein could be detected in Western Blot compared to MT-2 cells (Figure 3H). Therefore, transient expression of Tax is only sufficient to induce *COL4A1* and *COL4A2* transcripts, but additional mechanisms are required to promote presence of COL4 protein.

### Tax Slightly Transactivates the Bi-Directional *COL4A1-/COL4A2-* Promoter

**FIGURE 4:**
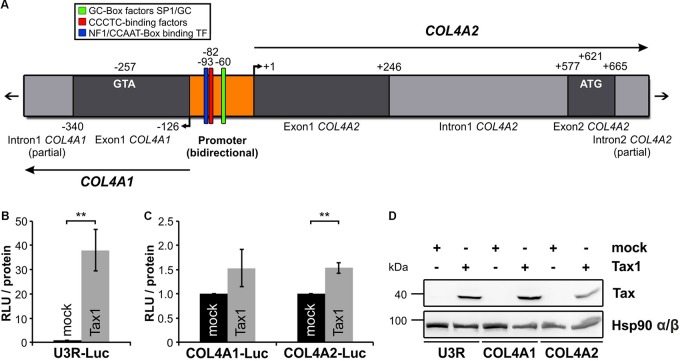
Tax transactivates the *COL4A1* and *COL4A2* promoter. **(A)** Schematic representation of the *COL4A1/2* genomic organization. In the shared and bidirectional *COL4A1/2* promoter region, putative genetic sites of GC-Box factors Specificity Protein 1 (SP1)/GC, CCCTC-binding factors and Nuclear Factor 1 (NF1)/CCAAT-Box binding transcription factors are indicated. Bioinformatic analysis was performed using the MatInspector software tool. **(B–D)** Conduction of luciferase-based reporter assays. **(B,C)** 5^∗^10^6^ Jurkat T-cells were co-transfected with a mock (pEFneo) or Tax expression vector (pEFneo-Tax1; 30 μg DNA) together with **(B)** a luciferase reporter vector for the U3R promoter from the HTLV-1 5′LTR region (U3R-Luc; 20 μg DNA) as a positive control, or **(C)** luciferase reporter vectors specific for the *COL4A1* or *COL4A2* promoter region (COL4A1-Luc, COL4A2-Luc; 20 μg DNA). Values were normalized on protein content and the luciferase background activity measured by co-expression of a promoter-less luciferase vector (pGL3-Basic). The mean of five independent experiments ± SE is depicted and Student’s t-test was performed (^∗∗^*p* < 0.01) comparing the different reporter vectors with their respective mock control. **(D)** Immunoblot staining of Tax and Hsp90 α/β protein served as expression control.

Expression of COL4 protein is not only tissue-dependent and typically absent in T-cells but is also highly regulated at several levels, including RNA stability, alternative splicing and posttranslational modifications (Khoshnoodi et al., 2008). Since COL4 expression is upregulated in HTLV-1-infected T-cells (Figures 1A–D) and Tax is sufficient to induce *COL4A1* and *COL4A2* transcripts (Figures 3A,B,D,E) we were interested in the question how Tax interferes with *COL4A1* or *COL4A2* regulation on genomic level. Therefore, we analyzed the short and bidirectional promoter of *COL4A1* and *COL4A2* for the presence of transcription factor binding sites by bioinformatics (Cartharius et al., 2005; Turner et al., 2015). We could not identify DNA sequences specific for promoter activation by Tax via the CREB pathway as were for example Tax responsive elements (TRE) (Shimotohno et al., 1986; Beimling and Moelling, 1992; Suzuki et al., 1993). However, we detected common and well described binding sites for transcription factors as Specificity Protein 1 (SP1) or Nuclear Factor 1 (NF1) (Figure 4A). Hence, we asked whether induction of *COL4A1* and *COL4A2* was due to indirect promoter activation by Tax. As a control, we made use of a luciferase-based reporter construct under the control of the *U3R*-fragment of the HTLV-1 promoter that is known to be transactivated by Tax via the CREB pathway (Xu et al., 1990; Mann et al., 2014). Upon co-transfection in Jurkat T-cells, Tax was able to significantly increase luciferase reporter activity by *U3R* promoter activation compared to an empty vector control as expected (Figure 4B). Performing the same experiment with luciferase-based reporter constructs specific for the *COL4A1* or the *COL4A2* promoter, co-expression of Tax in Jurkat T-cells in comparison to the respective empty vector control revealed that Tax augmented luciferase activity of the *COL4A1* and *COL4A2* reporter about 1.5-fold, even reaching significance for *COL4A2* (Figure 4C). This slight transactivation of the *COL4A1*/*COL4A2* promoter by Tax is supportive of a more complex regulation of type IV collagen gene expression. Tax protein expression during the reporter assays remained detectably constant and was checked by Western Blot analysis (Figure 4D). Summarizing, in spite of lacking Tax-specific binding sites, Tax is able to transactivate the *COL4A2* and, to a less extent, the *COL4A1* promoter potentially providing a mechanistic explanation for induction of *COL4A1* and *COL4A2* transcripts.

### Continuous Expression of Tax, but Not of the Herpesvirus Ateles Oncoprotein Tio, Is Necessary to Induce and Maintain COL4 Protein Expression

**FIGURE 5:**
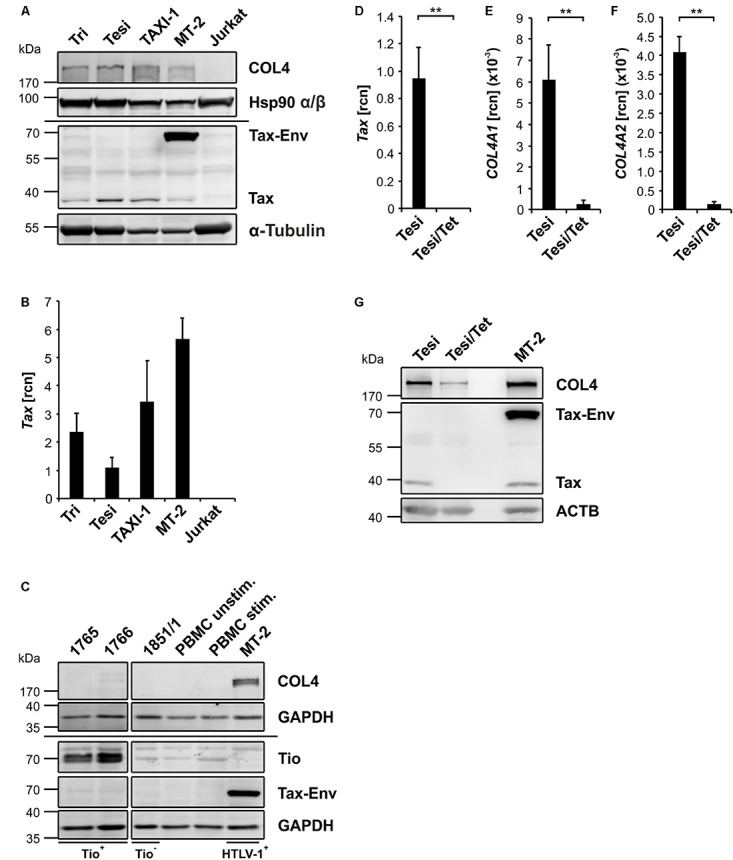
Continuous expression of Tax is necessary to induce and maintain COL4 protein expression. **(A,C)** Immunoblot of COL4 protein was carried out in **(A)** Tax-transformed T-cell lines (Tri, Tesi, TAXI-1) and **(C)** T-cell lines transformed by the oncoprotein Tio, encoded by Herpesvirus ateles (1765, 1766). The HTLV-1 positive T-cell line MT-2 served as positive control. In contrast, negative controls were the Tax and Tio negative T-cell lines Jurkat and 1851/1, as well as peripheral blood mononuclear cells (PBMC), that were either left unstimulated (unstim.) or stimulated (stim.) with 50 U/ml IL-2 together with 2 μg/ml PHA overnight followed by prolonged incubation with 50 U/ml IL-2 only. Staining of Tax, Tio and GAPDH were performed as control. **(B)** qPCR was performed to analyze abundance of *Tax* transcripts in Tri, Tesi, TAXI-1 and MT-2 T-cell lines. The mean relative copy numbers (rcn), normalized on *ACTB*, of one representative experiment ± SE are depicted. **(D–F)** Transcript levels of **(D)** *Tax*, **(E)** *COL4A1*, and **(F)** *COL4A2* were detected in the Tax-transformed T-cell line Tesi in the presence of Tax (Tesi) or after repression of Tax for 10 days by addition of 1 μg/ml tetracycline (Tesi/Tet). The mean relative copy numbers (rcn), normalized on *ACTB*, of three independent experiments ± SD are shown. Student’s *t*-test was conducted for statistical analysis (^∗∗^*p* < 0.01). **(G)** COL4 and Tax protein were detected in Tax-transformed T-cell lines expressing (Tesi) or repressing (Tesi/Tet) Tax protein and, as a control, in the HTLV-1 positive T-cell line MT-2. β-actin (ACTB) served as loading control.

Although Tax is able to induce *COL4A1* and *COL4A2* transcripts, transient expression of Tax was not sufficient to induce COL4 protein (Figures 3C,F). Thus, we asked whether an endured expression time of Tax protein leads to induction of COL4. As prolonged transient Tax expression in T-cells tends to lead to apoptosis after a certain period of time (Nicot and Harrod, 2000), we made use of the established Tax-transformed T-cell lines Tri, Tesi and TAXI-1 (Grassmann et al., 1992; Schmitt et al., 1998). These cell lines are derived from primary human T-cells immortalized by an expression cassette for Tax, which was transduced with a rhadinoviral vector (recombinant Herpesvirus saimiri). Compared to the control T-cell lines Jurkat (HTLV-1/Tax neg., COL4 neg.) and MT-2 (HTLV-1/Tax pos., COL4 pos.), robust expression of COL4 protein was a unique phenotype of the Tax-transformed T-cell lines Tri, Tesi and TAXI-1, suggesting that continuous expression of Tax can induce COL4 protein (Figure 5A). Due to different Tax protein expression levels in Tri, TAXI-1, and Tesi, varying from very low levels near the detection limit to strong, we also measured *Tax* transcripts confirming that all Tax-transformed cell lines express Tax although at different levels (Figure 5B). To exclude that T-cell transformation by any viral protein or the rhadinoviral vectors used to generate Tri, Tesi and TAXI-1 cells induces COL4 protein, we additionally analyzed CD4^+^ T-cell lines that were transformed by the Herpesvirus ateles oncoprotein Tio, which is - similar to Tax - a potent inducer of NF-*κ*B signaling (Heinemann et al., 2006; de Jong et al., 2010). For this purpose, we analyzed peripheral blood lymphocytes that were transduced by a Tio-expressing chimeric Herpesvirus saimiri resulting in the cell lines 1765 and 1766 (Albrecht et al., 2004) and the Tio-negative control cell line 1851/1 (Figure 5C). Compared to the Tio-, Tax- and COL4-negative cell line 1851/1 as well as primary stimulated and unstimulated PBMC, the HTLV-1 positive T-cell line MT-2 exhibited strong Tax-Env fusion and COL4 protein signals (Figure 5C). In contrast, albeit revealing clear Tio oncoprotein expression, none of the Tio-transformed cell lines 1765 and 1766 showed enhanced COL4 protein expression as MT-2 (Figure 5C). This finding further proved the uniqueness of Tax oncoprotein to induce COL4 protein in transformed T-cells and excluded that rhadinoviral Herpesvirus saimiri vectors induce COL4. To check whether Tax is important for maintenance of COL4 protein, we employed the Tesi/Tet cellular system, a Tax-transformed T-cell line with Tax expression (Tesi) being able to be repressed in presence of tetracycline (Tesi/Tet) (Schmitt et al., 1998). Tesi cells presented robust *Tax* transcript levels, which immediately dropped to almost zero after incubation with tetracycline indicating that the Tax repression system profoundly worked (Figure 5D). In the presence of Tax, *COL4A1* and *COL4A2* transcripts could readily be detected in Tesi cells. Intriguingly, repression of *Tax* in Tesi/Tet cells consecutively led to a nearly complete decline not only of *COL4A1* but also *COL4A2* transcripts (Figures 5E,F). In Western Blots, Tesi T-cells exhibited strong Tax as well as COL4 protein expression (Figure 5G). However, in Tesi/Tet, Tax protein almost vanished and COL4 protein decreased dramatically. Prolonged presence of Tax protein is therefore not only sufficient for induction of COL4 protein, but also necessary for maintenance in T-cells.

### COL4 and Gag p19 Partially Co-localize and Accumulate at the Virological Synapse and Can Be Transferred to HTLV-1 Negative T-cells in Co-culture

**FIGURE 6:**
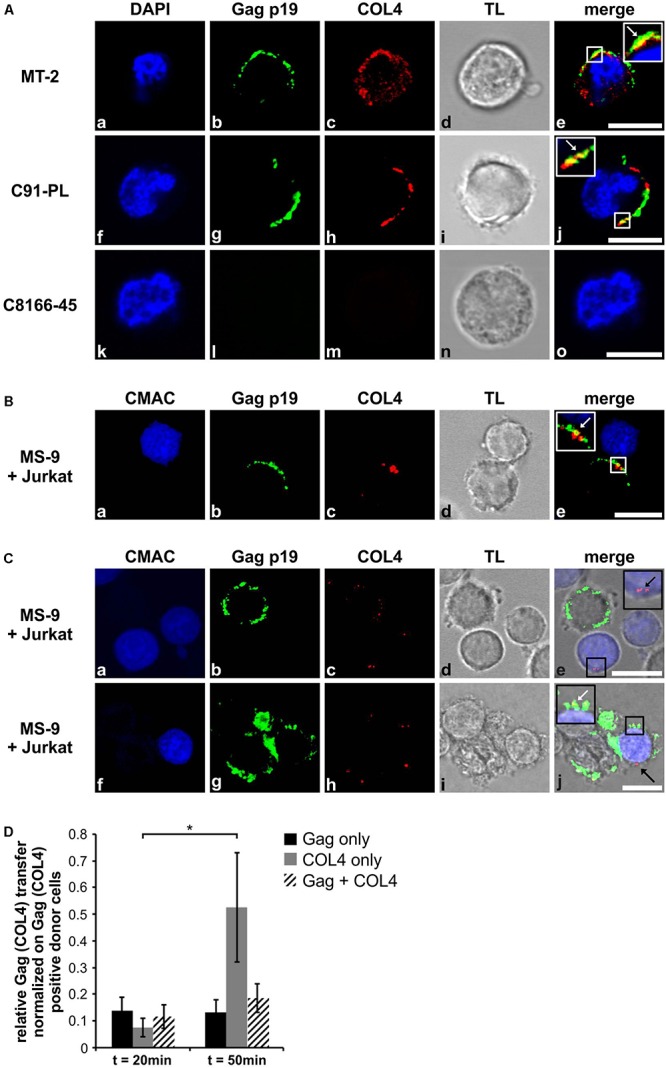
COL4 and Gag p19 partially co-localize and accumulate at the virological synapse and can be transferred to HTLV-1 negative T-cells in co-culture. **(Aa–o)** Immunofluorescence analysis was conducted in the HTLV-1 positive T-cell lines MT-2, C91-PL and C8166-45 spotted on poly-L-lysine coated coverslips. Permeabilized cells were stained with primary antibodies mouse anti-Gag p19 and the secondary antibody anti-mouse Alexa Fluor^®^ 488 (green), as well as rabbit anti-COL4 and the secondary antibody anti-rabbit Alexa Fluor^®^ 555 (red). DAPI staining of nuclei and transmitted light (TL) served as control. Insets show enlarged areas. A white arrow indicates partial co-localization of Gag and COL4 protein (yellow). The scale bar represents 10 μm. **(Ba–e,Ca–j)** Jurkat T-cells that had been prestained with CellTracker^TM^ Blue 7-Amino-4-Chlormethylcumarin (CMAC; 45 min, 20 μM, 37°C) were co-cultured with HTLV-1-infected MS-9 T-cells at a ratio of 1:1 for 20 or 50 min on poly-L-lysine coated coverslips. Following permeabilization, cells were stained with primary antibodies mouse anti-Gag p19 and the secondary antibody anti-mouse Alexa Fluor^®^ 488 (green), as well as rabbit anti-COL4 and the secondary antibody anti-rabbit Alexa Fluor^®^ 555 (red). Transmitted light served as control. Insets show enlarged areas. **(Ba–e)** A white arrow indicates partial co-localization of Gag and COL4 protein (yellow). **(Ca–j)** Black arrows indicate transferred COL4 protein from HTLV-1 positive MS-9 to Jurkat T-cells, a white arrow indicates partial co-localization of transferred Gag and COL4 protein (yellow). The scale bars represent 10 μm. **(D)** Manual counting was performed to quantify Gag and COL4 transfer from MS-9 donor cells to Jurkat acceptor cells. Gag only (black bars), COL4 only (gray bars) or Gag and COL4 (hatched bars) double positive Jurkat cells after 20 or 50 min of co-culture were determined and normalized on the number of Gag and/or COL4 positive MS-9 donor cells and on the ratio of Jurkat acceptor to MS-9 donor cells. Average values of counted cells ± SE are shown and Student’s *t*-test was conducted for statistical analysis (^∗^*p* < 0.05).

Since COL4 is upregulated in productively HTLV-1-infected T-cells, we next checked on implications of COL4 induction on the VB. We performed confocal laser scanning microscopy analyzing distribution of COL4 and Gag protein in the HTLV-1 positive T-cell lines MT-2 and C91-PL (Figure 6A), which had been spotted on poly-L-lysine coated coverslips. C8166-45 cells, which do not produce viral particles (Bhat et al., 1993), served as negative control for Gag and COL4 (Figures 1A–D). We found that COL4 (red) is predominantly expressed near or at the plasma membrane (Figures 6Ac,h), and dot-like spread all over the cell (Figure 6Ac). Moreover, Gag (green), as detected by Gag p19-specific antibodies, is strongly expressed in MT-2 and C91-PL cells and localizes to the plasma membrane (Figures 6Ac,h), while it is absent in C8166-45 cells (Figure 6Am). A partial overlap between Gag and COL4 fluorescence signals (yellow) could be observed as indicated by white arrows (Figures 6Ae,j, inset). For further analysis in more detail, MT-2 cells expressing huge amounts of Gag protein were not suitable. Therefore, we switched the cellular system to MS-9 cells, which is an HTLV-1 immortalized T-cell line containing only one proviral genome, thus expressing reasonable amounts of Gag protein (Shuh et al., 1999). To analyze involvement of COL4 in VB formation during formation of the VS and virus transfer, we performed confocal laser scanning microscopy of MS-9 cells in co-culture with Jurkat T-cells (Figure 6B). The latter cell line was pre-stained with CellTracker^TM^ Blue CMAC (7-Amino-4-Chlormethylcumarin; CMAC) as described previously (Donhauser et al., 2018) to discriminate between uninfected Jurkat T-cells (blue) and infected (non-blue) MS-9 cells. Briefly, CMAC is a dye that turns membrane-impermeable having crossed the plasma membrane. Cells were co-cultured (ratio 1:1) for 30 min at 37°C, spotted on poly-L-lysine coated coverslips, fixed and stained. Interestingly, examination of cell–cell contacts between infected MS-9 donor cells and Jurkat acceptor cells, stained in blue, revealed that the virological synapse was formed concentrating both COL4 (red) as well as Gag protein (green; detected with Gag p19-specific antibodies) toward the uninfected Jurkat T-cell (Figure 6B) in 50% of all VS detected. Further, Gag accumulated in clusters at the VS which are reminiscent of VBs (Pais-Correia et al., 2010), and even a partial overlap between Gag and COL4 protein could be observed as a yellow fluorescence signal at the virological synapse as indicated by a white arrow (Figure 6Be, inset). The partial co-localization was better determined by defining a region of interest (ROI) covering overlapping fluorescence signals which proved to have similar distributions (Supplementary Figure 4). Thus, COL4 as a component of the extracellular matrix may provide the scaffold for the attachment and concentration of budding viral particles into the VB. Going further, we were not only able to detect COL4 protein on HTLV-1 positive T-cells oriented toward target cells but we could also see, in some cases, patches of COL4 being transferred to HTLV-1 negative Jurkat acceptor cells (Figure 6C). In two examples, co-cultured MS-9 donor and Jurkat target cells are depicted showing parts of COL4 protein located on pre-stained COL4 negative Jurkat T-cells as indicated by black arrows (Figure 6Ce, inset j). Moreover, not only transferred COL4 but also Gag protein could be visualized presumably indicating transfer of VBs (Figure 6Cj, white arrow). Quantitative evaluation of imaging data revealed that after 20 min of co-culture, transfer of Gag could be observed in 14% of co-cultured Jurkat T-cells, suggesting that these cells are HTLV-1-infected (Figure 6D, black bars). A similar proportion of Jurkat T-cells (12%) received both Gag and COL4 (Figure 6D, hatched bars). Over time (*t* = 50 min), the proportion of cells receiving only Gag did not increase (13%), however, the frequency of Gag-positive cells receiving both Gag and COL4 slightly increased to 19% (*p* > 0.05), suggesting that receiving COL4 may be advantageous for getting HTLV-1-infected after pro-longed co-culture. Since we also observed patches of COL4 in Jurkat T-cells following co-culture with HTLV-1-infected MS-9 cells (Figure 6C), we next quantitated the frequency of COL4 transfer (Figure 6D). After 20 min of co-culture, 8% of co-cultured Jurkat T-cells received COL4 protein only (Figure 6D, gray bars). Interestingly, with a prolonged co-culture of 50 min, the frequency of COL4-positive Jurkat T-cells significantly increased to 53% (Figure 6D, gray bars) suggesting that transfer of COL4 between HTLV-1-infected cells to uninfected cells also occurs independent of virus transfer and dominates over the transfer of Gag at late time points post co-culture. Gag transfer appears to take place and be saturated very fast upon formation of cell-cell contacts whereas transfer of COL4 seems to be critical at later time points, indicating that Gag and COL4 exert mutual but also separate functions. Thus, we propose that COL4 protein plays a role not only in formation of the VS by being concentrated toward the cell–cell contact but that COL4 is also involved in formation of the VB and transfer to HTLV-1 negative target cells.

### Repression of COL4 Protein in Chronically Infected C91-PL Cells Impairs HTLV-1 Transfer to Co-cultured T-cells

**FIGURE 7:**
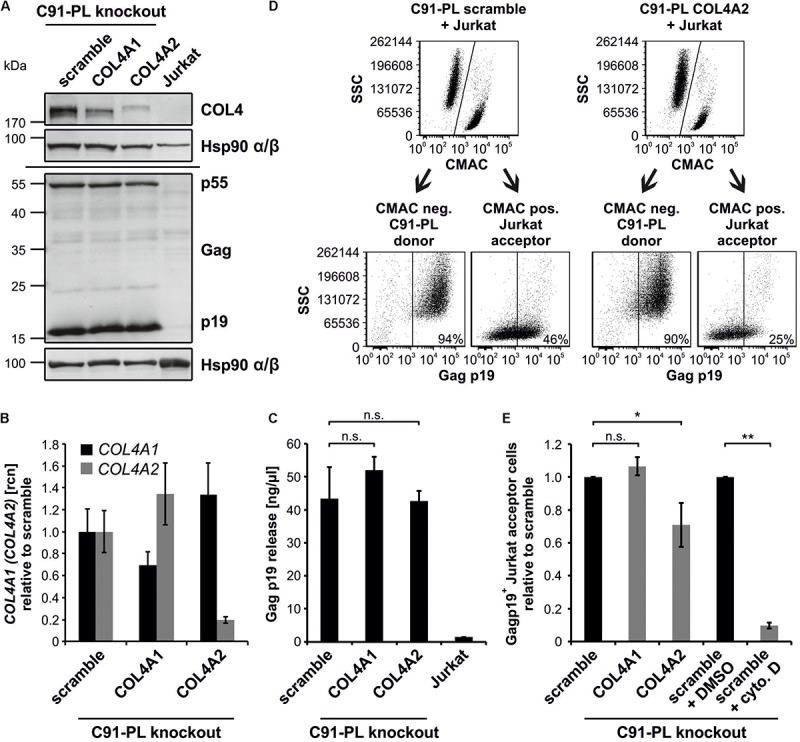
Repression of COL4 protein impairs virus transmission to uninfected T-cells, but not Gag processing and virus release in chronically infected C91-PL cells. **(A)** Western Blot analysis to detect COL4 as well as Gag p55 precursor and processed Gag p19 matrix protein was carried out in C91-PL cells stably transduced either by a CRISPR control vector (scramble) or vectors to generate a knockout for *COL4A1* or *COL4A2*. Hsp90 α/β served as loading control, lysates of Jurkat T-cells as negative staining control. **(B)** qPCR was performed to determine expression levels of *COL4A1* and *COL4A2* mRNA in transduced C91-PL cell lines from **(A)**. The mean relative copy numbers (rcn), normalized on *ACTB* and the C91-PL scramble control cell line, of one representative experiment ± SD are shown. **(C)** The amount of Gag p19 protein in the supernatant of transduced C91-PL cell lines from **(A)** was assessed by Gag p19 ELISA. The mean of five independent experiments ± SD is depicted and was compared using Student’s *t*-test (n.s.; not significant). Jurkat T-cells were employed as negative control. **(D,E)** Jurkat T-cells, that had been prestained with CellTracker^TM^ Blue 7-Amino-4-Chlormethylcumarin (CMAC), were co-cultured with stably transduced C91-PL cell lines from **(A)** at a ratio of 1:1 for 1 h. The transfer of Gag p19 to Jurkat acceptor cells was measured by flow cytometry using the primary antibody mouse anti-gag p19 and the secondary antibody anti-mouse Alexa Fluor^®^ 647. **(D)** The representative gating strategy for co-cultures of scramble and COL4A2 knockout C91-PL with Jurkat cells reveals discrimination between CMAC-positive Jurkat acceptor T-cells and CMAC-negative C91-PL donor cells, further detecting Gag p19 in the distinct cell populations. **(E)** The amount of Gag p19 positive Jurkat acceptor cells normalized on the scramble C91-PL control cells of three independent experiments ± SD is depicted and was compared using Student’s *t*-test (n.s., not significant; ^∗^*p* < 0.05; ^∗∗^*p* < 0.01). Treatment of C91-PL scramble cells with 5 μM cytochalasin D (cyto D), an inhibitor of actin-polymerization, in comparison to the DMSO solvent control served as positive control for an impaired Gag p19 transfer.

To study the impact of COL4 on HTLV-1 cell-to-cell transmission, we generated COL4 knockouts in the chronically HTLV-1-infected T-cell line C91-PL (Figure 7). This cell line expresses high amounts of COL4 (Figure 1D), partially co-localizing with HTLV-1 Gag (Figure 6Aj), and produces infectious viruses and VB (Alais et al., 2015). Briefly, we stably transduced C91-PL cells with lentiviral CRISPR vectors carrying either guide RNAs targeting *COL4A1*, *COL4A2* or a *scrambled* guide RNA (scramble). First, we verified the knockout of COL4 in C91-PL cells by western blot and qPCR. We could show that targeting *COL4A2* resulted in a sharp decrease of COL4 protein and *COL4A2* mRNA compared to control cells (scramble), while targeting *COL4A1* had only little impact on COL4 expression (Figures 7A,B). Interestingly, repression of COL4 did neither impair expression of Gag p55, processing to Gag p19 (Figure 7A), nor release of Gag p19 (Figure 7C) in chronically infected C91-PL cells. Since measuring of virus release by ELISA only quantitates virus-like particles and not necessarily infectious particles, we decided to measure HTLV-1 cell-to-cell transmission also directly by a flow cytometry based assay that allows monitoring of Gag transfer to target cells (Chevalier et al., 2014; Gross et al., 2016). For this purpose, we co-cultured C91-PL cells with uninfected Jurkat T-cells that had been pre-stained with CellTracker^TM^ Blue CMAC, which is retained in living cells for several generations and transferred to daughter cells, but not to neighboring cells (King et al., 2004; Donhauser et al., 2018). After 1 h of co-culture, cells were stained for Gag using anti-Gag p19 antibodies and flow cytometry was performed to discriminate cells according to the CMAC-staining into CMAC-negative donor cells (C91-PL) and CMAC-positive acceptor cells (Jurkat) and to determine the number of newly infected, Gag p19-positive Jurkat T-cells (Figure 7D). Flow cytometry revealed that repression of COL4A2 in C91-PL cells led to a significant reduction of HTLV-1 transfer to target cells as Gag p19-positive Jurkat T-cells were only 71% compared to the scramble control (Figure 7E). C91-PL cells pre-treated with cytochalasin D, which impairs actin-polymerization and thus, virus transmission, served as assay control (Figure 7E). Overall, our data show that COL4 affects Gag transfer between chronically infected T-cells and acceptor T-cells, while release of virus-like particles is unaffected by COL4 in this cell system, indicating an important role of COL4 in HTLV-1 cell-to-cell transmission.

## Discussion

HTLV-1 spreads at the virological synapse (VS), a virus-induced specialized cell-cell contact, by polarized budding into synaptic clefts (Igakura et al., 2003), and by cell surface transfer of viral biofilms (VBs) (Pais-Correia et al., 2010). In this study we shed further light on the role of the regulatory protein Tax in formation of the VB. We report that collagen IV (COL4), especially the collagen chains COL4A1 and COL4A2, are not only structural components of the basement membrane, but are also part of the VB and might thus be important for anchoring of HTLV-1 virions to the infected cell and for HTLV-1 transmission. Our study extends earlier work by Pais-Correia et al. (2010), who found collagens enriched in the VB in HTLV-1-infected cells, but randomly distributed in uninfected cells using pan-collagen antibodies, which recognize collagens 1–5. We specified in this study that COL4 is part of the VB and regulated by Tax. However, in our study using the same antibodies as Pais-Correia et al. (2010) (Supplementary Figure 2), we could not detect any COL1-5-specific signal in uninfected Jurkat T-cells, but only in HTLV-1-infected T-cells. Using COL4-specific antibodies we could neither detect COL4 in uninfected Jurkat T-cells nor in PBMCs, but only in the presence of HTLV-1 infection. Thus, it cannot be excluded that Pais-Correia detected additional collagens, like Collagen 1 in their study (Pais-Correia et al., 2010), which is detected by the pan –collagen antibody and whose alpha1 chain had been described to be transcriptionally induced by Tax in earlier work (Munoz et al., 1995) and in our microarray (Table 1).

Having found that COL4 upregulation is a unique feature of HTLV-1-infected cells with exception of the Rex-deficient cell line C8166-45 (Bhat et al., 1993), we searched for the mechanism of COL4 upregulation. In contrast to a variety of transformed B- and T-cell lines, COL4 protein was only upregulated in presence of continuous Tax-expression in either HTLV-1-infected or Tax-transformed T-cell lines suggesting that Tax is sufficient to induce COL4 expression, with the exception of C8166-45 cells. Thus, it cannot be excluded that another viral protein or a host factor that is absent in C8166-45 cells co-regulates COL4 expression together with Tax. Our study argues against a role of the viral Rex protein in regulating COL4 expression since co-expression of Rex-GFP and Tax did not further enhance *COL4A1* and *COL4A2* transcripts nor did it induce COL4 protein. Indeed, we found that Tax alone is sufficient to induce *COL4A1* and *COL4A2* transcripts, while robust induction of COL4 protein required continuous Tax expression. Tax’s moderate capacity to trans-activate the bi-directional *COL4A1*/*COL4A2* promoter is reminiscent of earlier work, which has shown that activity of this promoter is controlled by additional regulatory elements present on distant portions of both *COL4A1* and *COL4A2* genes as well as the presence of an enhancer within the first intron of *COL4A2* (Poschl et al., 1988; Pollner et al., 1990). Further, it is conceivable that Tax may affect COL4 expression at a post-transcriptional level since copy numbers of *COL4A1* and *COL4A2* are very low despite high levels of COL4 protein expression in HTLV-1-transformed cells and since transient expression of Tax alone is not sufficient to induce COL4 protein. However, our data using rhadinoviral-transformed T-cell lines expressing Tax clearly shows that COL4 protein is upregulated following continuous expression of Tax. Upregulation of COL4 does not seem to be a general feature of rhadinoviral-transformed T-cells like the Tax-expressing cell lines Tri, Tesi or TAXI-1 (Grassmann et al., 1992; Schmitt et al., 1998), as T-cells transformed with the tumorigenic rhadinoviruses Herpesvirus saimiri encoding the oncoproteins StpC/Tip, or encoding the Herpesvirus ateles oncoprotein Tio did not express COL4 similar to uninfected PBMC.

COL4 is the most important scaffold for the basement membrane and plays an important role in regulating tumor invasion and metastasis (Leitinger, 2011). It may also be advantageous for Tax-transformed T-cells, and potentially for ATLL-cells, too, to upregulate COL4. HTLV-1 may have evolved a mechanism to protect the infected and transformed cell by a collagen shield from adverse effects associated with the disease. Further, COL4 might be important for cell-cell-communication and for interaction with the tumor microenvironment by interactions between COL4 and its various receptors (Leitinger, 2011). Despite the upregulation of certain matrix metalloproteinases (MMP), MMP2 and MMP9 (Mori et al., 2002), which are unique type of proteinases that hydrolyze type IV collagen (Salo et al., 1983), we found upregulation of COL4 in HTLV-1-infected cells. Since COL4 plays an important role in regulating metastasis, the interplay between MMP activity and COL4 expression might be tightly regulated. Finally, HTLV-1/Tax may have evolved mechanisms to protect the transformed cells from apoptosis by upregulation of COL4. Earlier work has shown that collagens have a pro-survival and anti-apoptotic function in virus-transformed tumor cells by binding to their receptor DDR1 (Cader et al., 2013), which results in JNK-, ERK- and p38 MAPK-signaling (Leitinger, 2011).

Our data obtained in cell culture have also suggested a role of upregulated COL4 protein levels for VB formation *in vitro* and this may also be true for infected patients. Tax is necessary for COL4 maintenance *in vitro*. In primary cells derived from HAM/TSP patients, Tax expression is very low. Thus, detection of COL4 in patients would require higher numbers of Tax-expressing cells than usually observed. Since Tax is expressed in bursts and in individual cells (Billman et al., 2017; Mahgoub et al., 2018), it would be interesting to see, whether this transient expression of Tax *in vivo* is sufficient to upregulate COL4. Further, it remains to be determined whether COL4 is upregulated in ATLL patients where it could protect the infected tumor cell or may be important for interaction with the tumor microenvironment. Importantly, we found that *COL4A2* is upregulated in slowly progressing TSP/HAM patients suggesting that COL4A2 might be necessary for virus transmission *in vivo*.

COL4 is not only a structural component of the basement membrane, but also serves additional important functions like binding to other matrix proteins and to cells, anchoring to basement membrane (Tryggvason et al., 1990), and, as our work suggests, potentially also anchoring of HTLV-1. Having found that COL4 and the viral Gag p19 accumulate and co-localize in clusters at the VS and that COL4 and Gag are transferred to uninfected target cells, we hypothesize that COL4 is part of the VB and may be important for tethering of HTLV-1, and thus, HTLV-1 cell-to-cell transmission. This led us to the assumption that repression of COL4, and thus, interference with the VB, leads to an increase of HTLV-1 release similar to earlier work using heparin to disrupt the VB (Pais-Correia et al., 2010). However, against expectation, knockout of COL4 did not alter release of virus-like particles and HTLV-1 Gag processing in chronically infected C91-PL cells. Therefore, the interplay between COL4, its receptors on T-cells and potential virus-anchoring factors like BST-2 has to be further analyzed. Interestingly, HTLV-1 cell-to-cell transmission to co-cultured target T-cells was significantly impaired upon knockout of *COL4A2* in C91-PL cells. Since C91-PL cells form an infectious VB (Alais et al., 2015), our data suggest that COL4 may be important for cell-to-cell transmission of the VB. Of note, targeting *COL4A2* by gene editing strategies was more efficient than targeting *COL4A1* to repress COL4 protein expression in C91-PL cells, which may be due to the regulatory role of the COL4A2 chain in directing chain composition in the assembly of COL4A1-COL4A1–COL4A2 triple-helical molecules (Khoshnoodi et al., 2006). Thus, future studies should aim to repress COL4A1 protein as well to further dissect the distinct roles of COL4A1 and COL4A2 in COL4 protein formation and downstream functions.

Our observations showing that virus transmission is reduced upon repression of COL4 in C91-PL cells is indirectly supported by imaging of COL4 and Gag transfer between chronically infected MS-9 cells and Jurkat T-cells, indicating that COL4 seems to be important for tethering and cell-to-cell transfer of HTLV-1. Further, our data suggesting that COL4 is indeed part of the VB, and is transferred to uninfected target cells together with Gag are in line with earlier work, which has shown that extracellular matrix components are transferred together with viral components to target cells (Pais-Correia et al., 2010). However, our data specify that COL4 is transferred to target cells and that this may also happen independent of HTLV-1 Gag transfer, which is reminiscent of a mechanism termed trogocytosis. During trogocytosis, two cells form a transient intimate interaction during which the membranes appear to fuse and plasma membrane proteins are exchanged between the two cells. This mechanism is e.g., exploited for bacterial transfer when host cells exchange plasma membrane proteins and cytosol via a trogocytosis-related process, which leaves both donor and recipient cells intact and viable (Steele et al., 2016). Thus, further work is needed to clarify the role of collagen transfer between cells and the mechanism how COL4 affects HTLV-1cell-to-cell transmission.

## Conclusion

In conclusion, our study identifies the extracellular matrix protein COL4 as a novel target of Tax and a component of the viral biofilm (VB), and might thus contribute to a better understanding of cellular processes regulating not only VB formation, but also virus transmission.

## Data Availability Statement

The microarray data have been deposited in NCBI’s Gene Expression Omnibus (Edgar et al., 2002) and are accessible at http://www.ncbi.nlm.nih.gov/geo and GEO Series accession numbers are GSE10508 and GSE17718 (https://www.ncbi.nlm.nih.gov/geo/query/acc.cgi?acc=GSE10508; https://www.ncbi.nlm.nih.gov/geo/query/acc.cgi?acc=GSE17718).

## Ethics Statement

The studies involving human participants were reviewed and approved by Ethics Committee of the French Ministry of Research and Ethics Committee of the Medical Faculty of Friedrich-Alexander-Universität Erlangen-Nürnberg. Written informed consent for participation was not required for this study in accordance with the national legislation and the institutional requirements.

## Author Contributions

SM performed and designed the experiments, analyzed the data, and wrote the manuscript. CG performed and designed the experiments, and analyzed the data. ND performed the experiments and analyzed the data. MM performed the experiments. J-MP performed the experiments and analyzed the data. AT-K conceived of the study, designed the experiments, analyzed the data, and wrote the manuscript.

## Conflict of Interest

The authors declare that the research was conducted in the absence of any commercial or financial relationships that could be construed as a potential conflict of interest.
