# Supplementary material for: Collagen IV (COL4A1, COL4A2), a Component of the Viral Biofilm, Is Induced by the HTLV-1 Oncoprotein Tax and Impacts Virus Transmission
Source: Front Microbiol. 2019 Oct 23;10:2439. doi: 10.3389/fmicb.2019.02439 (PMC6819499; doi:10.3389/fmicb.2019.02439)
Supplement: Supplementary file 1 [file Data_Sheet_1.pdf]

Supplementary Material

1 Supplementary Figures and Tables

1.1 Supplementary Figures

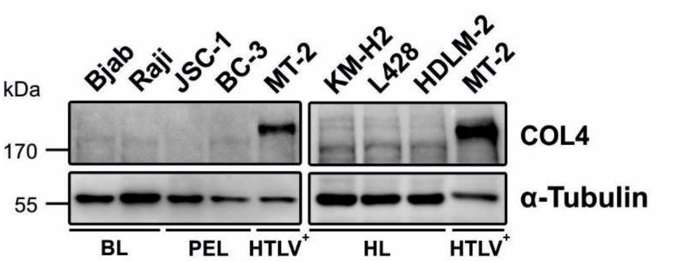

Supplementary Figure 1. COL4 protein is upregulated in HTLV-1 positive T-cells only. Immunoblotting shows COL4 protein expression in cell lines established from Burkitt lymphoma (BL; Bjab, Raji), Primary Effusion Lymphoma (PEL; JSC-1, BC-3) and Hodgkin Lymphoma (HL; KM-H2, L428, HDLM-2). Staining of COL4 protein in the HTLV-1 positive T-cell line MT-2 served as positive control, staining of α-Tubulin as loading control.

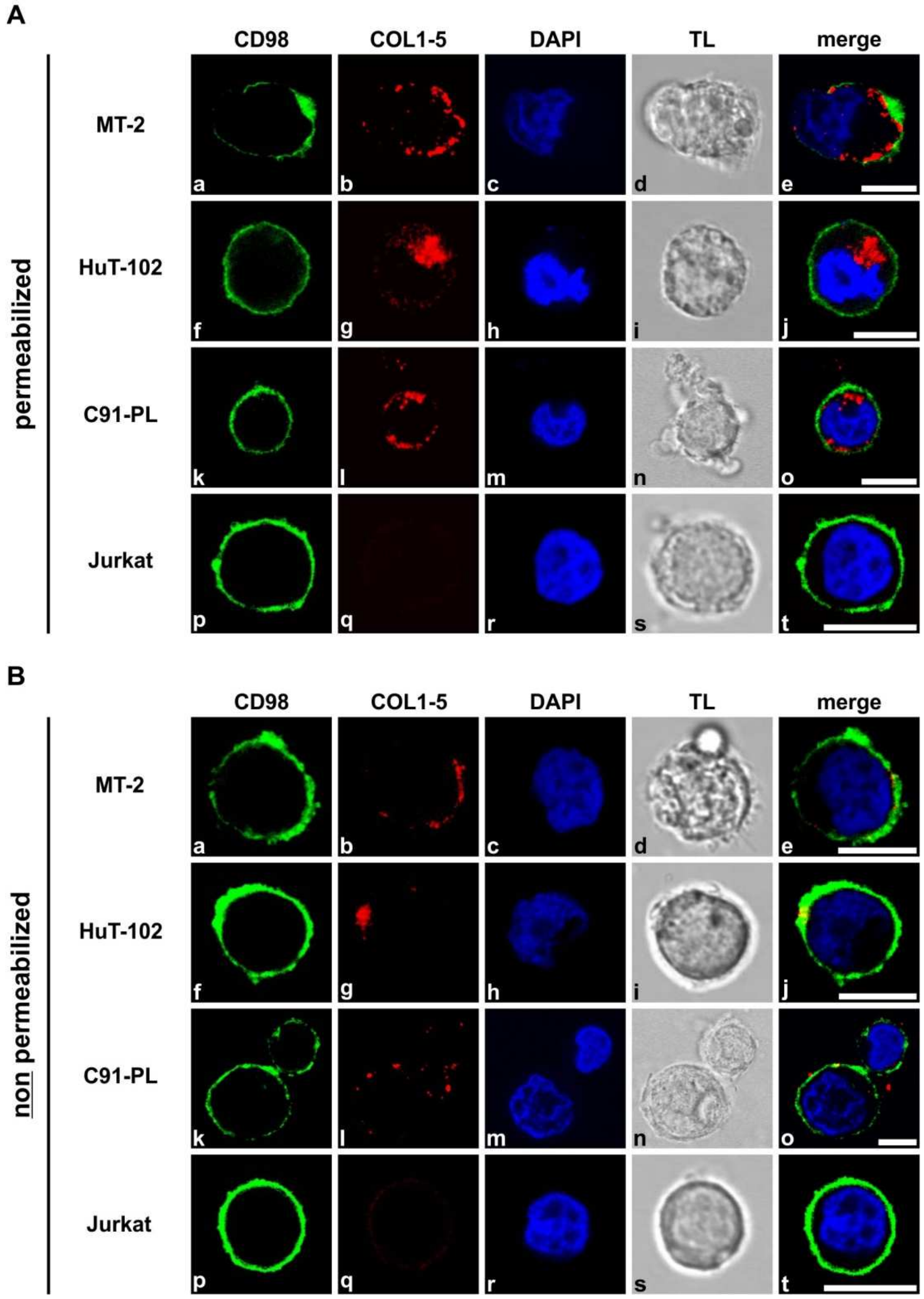

Supplementary Figure 2. COL1-5 protein localizes to extra- and intracellular compartments in HTLV-1 positive T-cells. (A, B) Confocal microscopy was performed in the HTLV-1 positive T-cell lines MT-2, HuT-102 and C91-PL and in the HTLV-1 negative T-cell line Jurkat. Cells were spotted on epoxy-resin coated coverslips and fixed, washed with PBS containing 0.1 % Tween®-20 and **(A)** were permeabilized by 0.2 % Triton™ X-100 for 20 min at 4 °C or **(B)** were left unpermeabilized by incubation with PBS for 20 min at 4 °C. Cells were washed twice and unspecific binding sites were blocked with PBS comprising 5 % FCS and 1 % BSA for 1 h at room temperature. Primary antibodies recognizing the plasma membrane marker CD98 (ab2528, abcam) or COL1-5 (2150-2206, BioRad) were applied for 45 min at 37 °C. Cells were washed three times and incubated with secondary

antibodies Alexa Fluor® 488 anti-mouse (CD98 in green) or Alexa Fluor® 647 anti-rabbit (COL1-5 in red) for 45 min at 37 °C one after the other with three washing steps in between. After three final washing steps, cells were fixed with *ProLong® Gold Antifade Mountant medium with DAPI* (Life Technologies) to stain the nuclei (blue). Images were acquired using a *Leica TCS SP5* confocal laser scanning microscope equipped with a 63x1.4 HCX PL APO CS oil immersion objective lens (Leica Microsystems). Images of transmitted light (TL) served as control. The scale bars represent 10µm.

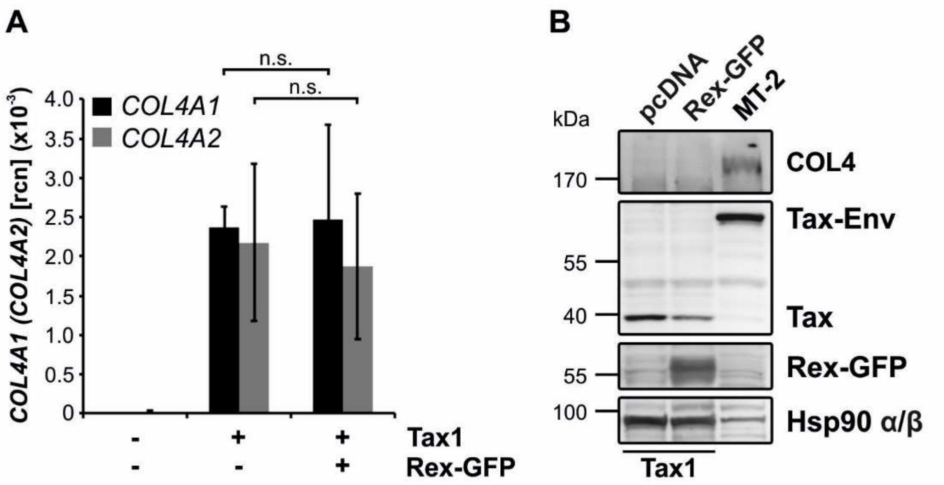

Supplementary Figure 3. Rex does not cooperate with Tax in regulating COL4 expression. (A, B) 10*10^6 Jurkat T-cells were co-transfected with a Tax expression vector (pEFneo-Tax1; 60 µg DNA) together with a mock (pcDNA) or Rex-GFP fusion protein expression vector (pCMV-Rex1-GFP; 40 µg). **(A)** *COL4A1* and *COL4A2* transcript levels were quantified by qPCR in untreated or Tax and Rex-GFP transfected Jurkat T-cells. The mean relative copy numbers (rcn), normalized on *ACTB*, of three independent experiments ± SD are depicted. Student's t-test was conducted for statistical analysis (n.s., not significant). **(B)** Western Blot analysis was carried out staining COL4, Tax, Rex-GFP protein (using GFP-specific antibodies), and Hsp90 α/β as loading control. Detection of COL4 and Tax protein in the HTLV-1 positive T-cell line MT-2 served as positive control.

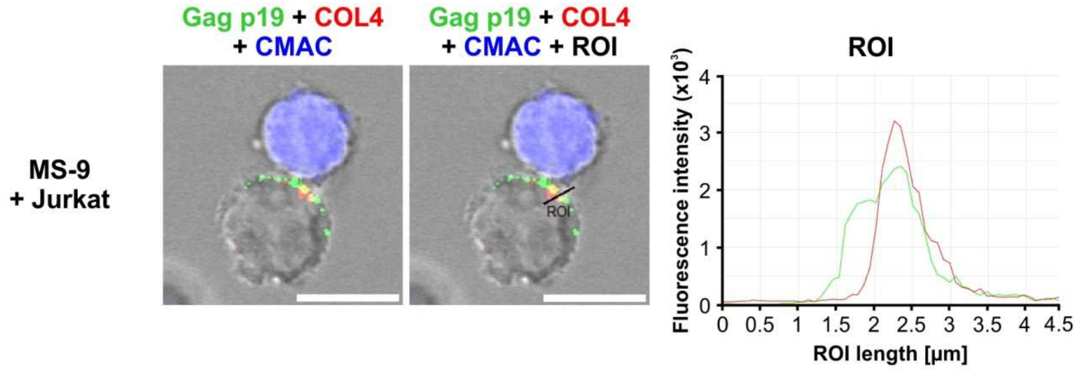

Supplementary Figure 4. COL4 and Gag p19 partially co-localize and accumulate at the virological synapse. HTLV-1 negative Jurkat T-cells were prestained with the cell-permeable dye *CellTracker™ Blue 7-Amino-4-Chlormethylcumarin* (CMAC; Thermo Fisher Scientific; 45 min, 20 µM, 37 °C; blue). Pre-stained Jurkat and HTLV-1 positive MS-9 cells were co-cultured for 30 min at 37 °C at a ratio of 1:1 on poly-L-lysine coated coverslips. Cells were fixed in 2 % PFA for 1 h at room temperature and after five washing steps with PBS containing 0.1 % Tween®-20, cells were permeabilized by 0.2 % Triton™ X-100 for 20 min at 4 °C. Cells were washed twice and unspecific binding sites were blocked with PBS comprising 5 % FCS and 1 % BSA for 1 h at room temperature. Primary antibodies recognizing Gag p19 (mouse; TP-7, Zeptometrix) or COL4 (rabbit; ab6586, abcam) were applied for 45 min at 37 °C, followed by three washing steps. Cells were incubated with secondary antibodies Alexa Fluor® 488 anti-mouse (Gag p19 in green) and Alexa Fluor® 647 anti-rabbit (COL4 in red) for 45 min at 37 °C one after the other with three washing steps in between. After three final washing steps , cells were fixed with *ProLong® Gold Antifade Mountant medium without DAPI* (Life Technologies). Images were acquired using a *Leica TCS SP5* confocal laser scanning microscope equipped with a 63x1.4 HCX PL APO CS oil immersion objective lens (Leica Microsystems). A merge of CMAC (blue), Gag p19 (green) and COL4 (red) stain together with transmitted light is depicted. Images were analyzed using the LAS AF software (Leica Microsystems): A region of interest (ROI) was defined showing the corresponding signals of Gag p19 and COL4 fluorescence intensities. The scale bars represent 10 µm.

1.2 Supplementary Tables

Supplementary Table 1.

Transcriptional expression of components of the viral biofilm.

	average signals[a]				fold change[b]				
	Tax-positive		Tax-negative						
Gene	MT-2	Tesi	$CD4^+$	Tesi/Tet	MT-2 vs $CD4^+$	p value	Tesi vs Tesi/Tet	p value	probe set
**AGRIN**	68	150	65	122	1	0.79	1	0.13	212285_s_at
**AGRIN**	51	90	52	54	-1	0.97	2	0.41	217419_x_at
**AGRIN**	(5)	(6)	(6)	(9)	n.a.		n.a.		217410_at
**AGRIN**	(8)	(8)	(11)	23	-1	0.82	-3	0.04	212283_at
**BST2**	65	59	29	73	2	0.09	-1	0.02	1570198_x_at
**BST2**	39	49	(11)	32	4	0.12	2	0.09	1570197_at
**BST2**	468	730	516	650	-1	0.29	1	0.51	201641_at
**COL1A1**	263	161	(2)	65	272	0.06	2	0.17	1556499_s_at
**COL1A1**	108	53	(4)	28	38	0.04	2	0.27	202310_s_at
**COL1A1**	(12)	(4)	(5)	(17)	n.a.		n.a.		202311_s_at
**COL1A1**	(9)	(15)	(12)	(16)	n.a.		n.a.		202312_s_at
**COL1A1**	64	46	41	51	2	0.41	-1	0.89	217430_x_at
**COL1A2**	(2)	(2)	(2)	(2)	n.a.		n.a.		229218_at
**COL1A2**	(4)	(8)	(2)	(1)	n.a.		n.a.		202404_s_at
**COL1A2**	(3)	(13)	(18)	(17)	n.a.		n.a.		202403_s_at
**COL2A1**	(5)	(13)	(10)	(11)	n.a.		n.a.		213492_at
**COL2A1**	(4)	(5)	(1)	(2)	n.a.		n.a.		217404_s_at
**COL3A1**	(11)	1604	(5)	1614	n.a.		-1	0.99	215076_s_at
**COL3A1**	(6)	986	(3)	992	n.a.		-1	0.93	201852_x_at
**COL3A1**	(7)	492	(3)	449	n.a.		1	0.51	211161_s_at
**COL3A1**	(1)	57	(3)	70	n.a.		-1	0.14	232458_at
**COL4A1**	(7)	(13)	(2)	24	n.a.		-2	0.48	233652_at
**COL4A1**	1780	360	(16)	92	109	<0.01	4	<0.01	211980_at
**COL4A1**	855	128	(4)	40	267	0.02	3	<0.01	211981_at
**COL4A2**	1325	624	(4)	233	331	<0.01	3	0.06	211964_at
**COL4A2**	604	205	(8)	65	87	0.02	3	0.03	211966_at
**COL4A2**	33	(15)	(4)	(5)	10	0.03	n.a.		237624_at

Supplementary Table 1. continued

Gene	average signals[a]				fold change[b]				probe set
	Tax-positive		Tax-negative						
	MT-2	Tesi	$CD4^+$	Tesi/Tet	MT-2 vs $CD4^+$	p value	Tesi vs Tesi/Tet	p value	
**COL4A3**	(5)	(14)	(14)	(11)	n.a.		n.a.		216898_s_at
**COL4A3**	(1)	(7)	(1)	(7)	n.a.		n.a.		216896_at
**COL4A3**	(9)	(12)	(9)	(8)	n.a.		n.a.		216368_s_at
**COL4A3**	(3)	(7)	(2)	(11)	n.a.		n.a.		216367_at
**COL4A4**	(1)	(1)	(1)	(1)	n.a.		n.a.		214602_at
**COL4A4**	(10)	(3)	(1)	(4)	n.a.		n.a.		229779_at
**COL4A4**	(4)	(9)	(9)	(15)	n.a.		n.a.		241565_at
**COL4A5**	(2)	13	(1)	(14)	n.a.		-1	0.72	234387_at
**COL4A5**	(7)	(1)	(6)	(6)	n.a.		n.a.		213110_s_at
**COL4A5**	(8)	(9)	(6)	(8)	n.a.		n.a.		1563536_at
**COL4A6**	(14)	(16)	(2)	21	n.a.		-1	0.26	1564654_at
**COL4A6**	(7)	(3)	(7)	(6)	n.a.		n.a.		210945_at
**COL4A6**	(7)	(10)	(6)	(7)	n.a.		n.a.		213992_at
**COL4A6**	(6)	(5)	(3)	(2)	n.a.		n.a.		211473_s_at
**COL5A1**	(4)	(2)	(4)	(8)	n.a.		n.a.		212489_at
**COL5A1**	(5)	(18)	(11)	24	n.a.		n.a.		1556138_a_at
**COL5A1**	(2)	(2)	(4)	(2)	n.a.		n.a.		212488_at
**COL5A1**	(5)	(4)	(3)	(5)	n.a.		n.a.		203325_s_at
**COL5A2**	(8)	(4)	(18)	(8)	n.a.		n.a.		221730_at
**COL5A2**	(11)	(6)	(15)	(8)	n.a.		n.a.		221729_at
**COL5A3**	(12)	(4)	(15)	(4)	n.a.		n.a.		52255_s_at
**COL5A3**	(2)	(3)	(4)	(2)	n.a.		n.a.		218975_at
**FUT4**	(5)	(12)	(15)	(12)	n.a.		n.a.		244889_at
**FUT4**	(4)	(9)	(7)	(5)	n.a.		n.a.		1560995_s_at
**FUT4**	148	90	71	31	2	<0.01	3	0.03	209892_at
**FUT4**	98	38	61	31	2	0.32	1	0.37	209893_s_at
**FUT9**	(2)	(1)	(1)	(1)	n.a.		n.a.		207696_at
**FUT9**	(4)	(1)	(2)	(2)	n.a.		n.a.		216185_at
**LGALS3**	(4)	27	(6)	27	1	0.91	1	0.95	1557197_a_at
**LGALS3**	2043	4345	1014	4490	2	<0.01	-1	0.54	208949_s_at

[a] average signals were rounded to integers; values in brackets were deemed absent calls.

[b] values depicting the fold change of transcript expression were rounded to integers; p values were rounded on two decimals; vs, versus; n.a., not applicable.
